# Harnessing exosomal long non-coding RNAs as a new frontier for molecular diagnostics and therapeutics in diabetes mellitus

**DOI:** 10.1186/s12964-026-02739-w

**Published:** 2026-03-12

**Authors:** Heba M. Midan, Eman F. Wasfey, Ahmed S. Doghish, Mohamed M. Kamal, Dina H. Kassem

**Affiliations:** 1 Department of Biochemistry, Faculty of Pharmacy, Badr University in Cairo (BUC), Cairo, Badr City, 11829 Egypt; 2 Department of Biochemistry and Molecular Biology, Faculty of Pharmacy, Ain Shams University, Cairo, 11566 Egypt; 3 Department of Biochemistry and Molecular Biology, Faculty of Pharmacy (Boys), Al-Azhar University, Cairo, Nasr City, 11231 Egypt; 4 Department of Pharmacology and Biochemistry, Faculty of Pharmacy, The British University in Egypt, Cairo, Egypt; 5 The Centre for Drug Research and Development, Faculty of Pharmacy, The British University in Egypt, Cairo, Egypt

**Keywords:** Diabetes mellitus, Extracellular vesicles, Exosomes, Lnc-RNAs, Insulin resistance, β-cell dysfunction, And biomarkers

## Abstract

Exosomes are emerging as dynamic regulators of cellular crosstalk. Exosomal long non-coding RNAs (Exo-lncRNAs) are now recognized as key molecular mediators of diabetes mellitus. These molecules are interrelated with β-cell function, insulin resistance, inflammation, and diabetic complications. Key diabetes-related exo-lncRNAs include exo-P3134, which supports β-cell compensation, as well as exo-MALAT1 and exo-GAS5, which are regulators of insulin resistance and β-cell dysfunction. This review unravels the complex interplay between exo-lncRNAs and the hallmarks of diabetes, across disease pathogenesis and complications. It also spotlights the unprecedented potential of exo-lncRNAs as non-invasive biomarkers and next-generation therapeutic targets in precision medicine in diabetes. By combining mechanistic and translational evidence, this review offers the first integrative framework that positions exo-lncRNAs at the center of diabetes progression and opens new avenues for translational applications.

**Graphical Abstract:**

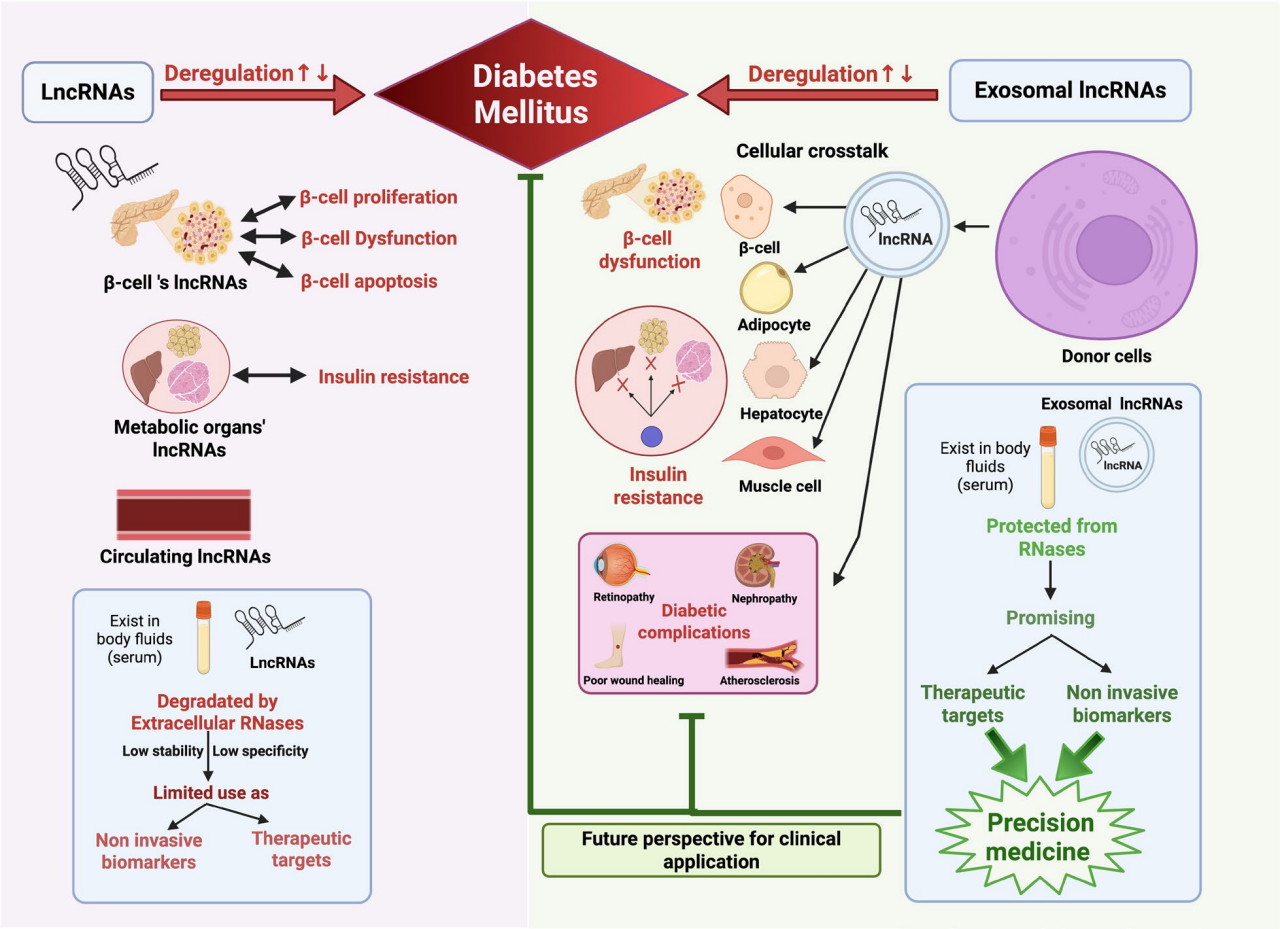

## Introduction

### Diabetes mellitus

#### The current landscape of diabetes mellitus worldwide

Diabetes mellitus (DM) has emerged as a major global health burden, with 589 million people affected in 2024, and approximately 3.4 million related deaths. Alarmingly, this prevalence is expected to continue rising, reaching almost 853 million people worldwide by 2050 [1].

Type 2 Diabetes Mellitus (T2DM) is the predominant form of diabetes, accounting for 90–95% of all diabetes cases worldwide [2]. Its pathogenesis is multifactorial and typically characterized by four major metabolic disturbances: impaired insulin action, dysfunction of pancreatic β-cells, excessive glucose production by the liver, and excess body fat [3–6]. Additionally, impaired glucose tolerance (IGT) is recognized as a critical prediabetic state that significantly elevates the risk of progressing to T2DM [7, 8]. The rising prevalence of T2DM is largely attributed to modifiable lifestyle factors, including increased rates of obesity, sedentary behavior, and unhealthy dietary patterns [2].

#### Problem statement

There is a silent gap in diabetes progression during the critical period of metabolic dysfunction that precedes the elevation of blood glucose, characterized by progressive decline in β-cell function [9, 10]. The traditional diagnostic methods, like fasting blood glucose and glycated hemoglobin, can detect overt hyperglycemia, but they may fail to capture the early stages of the disease, raising the need for sensitive early detection biomarkers [11, 12]. Furthermore, current pharmacological therapies remain insufficient to eliminate the burden of diabetes-related microvascular and macrovascular complications [13, 14]. In the subclinical phase, the body maintains euglycemia through compensatory hyperinsulinemia, which delays the onset of overt hyperglycemia. During this period, microvascular injury may have already initiated [15, 16]. Therefore, the development of sensitive biomarkers may help in identifying the transition from compensatory hyperinsulinemia to inadequate β-cell secretory function before diabetes becomes clinically apparent, as well as tailoring the patients’ pharmacological therapies based on their degree of β-cell dysfunction or insulin resistance [17]. Taken together, there is an urgent need for efficient molecular biomarkers to enable earlier diagnosis and individualized intervention. Among those molecular biomarkers, long non-coding RNAs (lncRNAs) have emerged as promising candidates to fulfil this role [18].

### Long non-coding RNAs

Long non-coding RNAs (lncRNAs) are a type of non-coding RNA composed of transcripts exceeding 200 nucleotides (nt). They were thought to be transcriptional noise as they lack the ability to be translated into proteins. However, they have been recognized to serve significant functions in post-transcriptional processes, chromatin remodeling, intracellular trafficking, and epigenetic control [19]. LncRNAs regulate a wide range of biological functions and disease processes, including their critical roles in diabetes development, where they maintain β-cell homeostasis, govern lipid metabolism, and control inflammation [20–23]. They are present in various bodily fluids, including serum, plasma, and urine. Correspondingly, lncRNAs have shown promise as diagnostic and prognostic indicators and as viable candidates for therapeutic targeting [24].

Interestingly, growing evidence indicates that specific lncRNAs are selectively incorporated into and transported by small extracellular vesicles known as exosomes [25]. Exosomal lncRNAs (exo-lncRNAs) offer several advantages over circulating lncRNAs. The lack of tissue specificity and susceptibility to degradation of circulating lncRNAs can limit their utility in monitoring disease progression [26, 27]. On the other hand, exosomes confer enhanced stability and protection for lncRNAs from degradation [27]. Additionally, exosomes carry surface proteins that identify their tissue of origin. In this context, exo-lncRNAs serve as messages of active cell-to-cell communication system, therefore, they provide a real-time image of the health of specific organs, i.e., exosomes derived from failing β-cell carry a different molecular signature than a general circulating RNA signature [28, 29]. Moreover, the circulating lncRNAs represent a mixture resulting generally from cells turn-over all over the body, however, exosomes are mostly enriched by high concentrations of specific lncRNAs, which are secreted by living cells, thereby enabling the diagnostic tools to detect a clean signal rather than the noisy signal of the circulating lncRNAs [26, 27].

### Exosomes

Exosomes are small extracellular vesicles ranging in size from 30 to 150 nm and are secreted by almost all cell types. They transport bioactive components, including lncRNA, microRNA (miRNA), messenger RNA (mRNA), and proteins, to the recipient cells [30], thus facilitating organ crosstalk and tissue-to-tissue signaling. These vesicles can be extracted from various biological fluids, including plasma and serum. Any change in exosome amount and composition may detect the onset and progression of different diseases [31]. Critically, exosomes and their active cargo, particularly exo-lncRNAs, can significantly influence pancreatic β-cell function, insulin secretion, and inflammation [32]. Consequently, exosomal lncRNAs combine the diagnostic potential of lncRNAs with the stability of vesicular transport, making them ideal candidates for the early diagnosis and therapeutic targeting of diabetes [33–35].

### Review methodological approach

An online literature search was conducted between September 2023 and December 2025 to identify scientific studies and diverse review papers published in English over the past 10 years. The search was conducted using electronic medical research databases, including ScienceDirect and PubMed. The subsequent keywords and combinations were used: “Diabetes Mellitus” OR “DM” AND “long non-coding RNAs” OR “lncRNAs” AND “exosomes” OR “exosomal lncRNAs” AND “insulin resistance” AND “diagnostic biomarker” AND “therapeutic target”; as well as “exosomal lncRNAs” AND “β-cell dysfunction”, and “exosomal lncRNAs” AND “diabetic complications.

### Review aim

In this narrative review, we investigate the emerging roles of exo-lncRNAs in diabetes, highlighting their potential role as biomarkers for early detection and as targets for treatment of diabetes and its complications. First, the review outlines the contribution of lncRNAs to the underlying mechanisms of diabetes, with particular focus on those expressed in pancreatic β-cells, metabolic tissues, and circulating compartments. Next, we provide an in-depth overview of current literature describing the involvement of exo-lncRNAs in the initiation and development of various forms of diabetes. The mechanistic interactions between exo-lncRNAs and critical molecular pathways underlying impaired insulin signaling and β-cell impairment are also explored. Furthermore, the prospective clinical utility of exo-lncRNAs in identifying, monitoring, and managing diabetes and its secondary effects are discussed. As a final point, we highlight the current obstacles, knowledge gaps, and prospective avenues of research essential for bringing exo-lncRNA-based strategies into clinical use.

## LncRNAs

### Key characteristics of LncRNAs

Among many non-coding RNAs, novel functional lncRNAs have emerged [36]. lncRNAs are characterized by polyadenylation, splicing signals, exons, and regulatory elements, and represent a major component of the mammalian noncoding RNA transcriptome [37].These transcripts, over 200 nucleotide long, are not translated into proteins and typically show more restricted, cell-specific expression than mRNAs, often localizing predominantly in the nucleus but also in the cytoplasm [19].

**Fig. 1:**
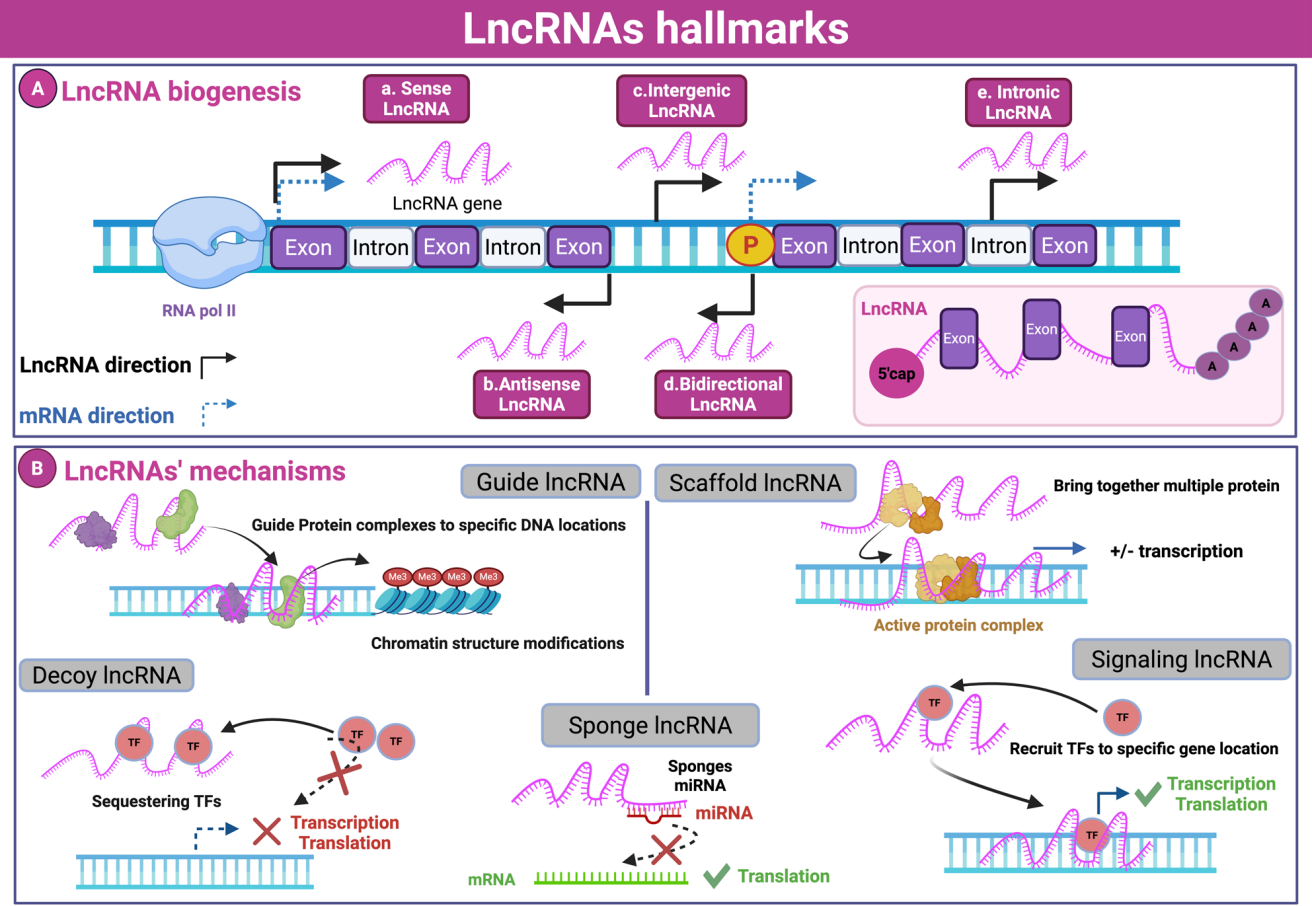
Biogenesis and molecular mechanisms of lncRNAs. (**A**) Biogenesis of lncRNAs, illustrating their classification into sense, antisense, bidirectional, intergenic, and intronic categories based on their transcriptional orientation and genomic location. (**B**) Functional mechanisms of lncRNAs include their roles as guides for chromatin modification, scaffolds for protein complexes, decoys for transcription factors, sponges for miRNAs, and signaling mediators for transcription regulation.*TF: transcription factor*. This figure was created in https://BioRender.com

LncRNAs are classified by their genomic position in relation to the adjacent protein-coding genes. In such a way, they fall into distinct 5 categories: sense, antisense, intronic, intergenic, and bidirectional lncRNAs [38]. Sense lncRNAs induce local epigenetic changes [39]; antisense lncRNAs regulate complementary transcripts [40]; intronic lncRNAs arise from introns [41]; intergenic lncRNAs are transcribed from noncoding genomic regions [42]; and bidirectional lncRNAs share promoters with nearby genes [43]. Biogenesis and various classifications of lncRNAs are summarized in Fig. 1A.

Functionally, lncRNAs serve diverse roles: they can guide protein complexes to specific genomic sequences for chromatin modification, act as decoys by sequestering transcription factors, and operate as sponges that competitively bind miRNAs to modulate post-transcriptional gene regulation [36]. Additionally, lncRNAs act as signaling regulators, recruiting transcription factors and influencing chromatin structure [19]. These overlapping roles demonstrate that one lncRNA can perform multiple functions dependent on cellular context [19, 36].

In short, lncRNAs modulate gene expression through interactions with DNA and mRNA inside the nucleus, as well as with miRNAs and proteins in the cytoplasm [44]. Various biological mechanisms of lncRNAs are demonstrated in Fig. 1B.

### LncRNAs as mediators of cellular communication in diabetes mellitus

Various inter-organ and inter-cellular crosstalk mechanisms have been reported to play pivotal roles in diabetes pathogenesis. For example, our lab reported previously that a hepatokine named serpin family B member 1 (SERPINB1) and an islet-secreted cytokine, pancreatic-derived factor (PANDER), have been found to be interrelated with β-cell dysfunction and poor glycemic control [45–47]. Likewise, we previously reported that vaspin and visfatin are adipokines interconnected with various deriving factors involved in T2DM pathogenesis [48].

**Fig. 2:**
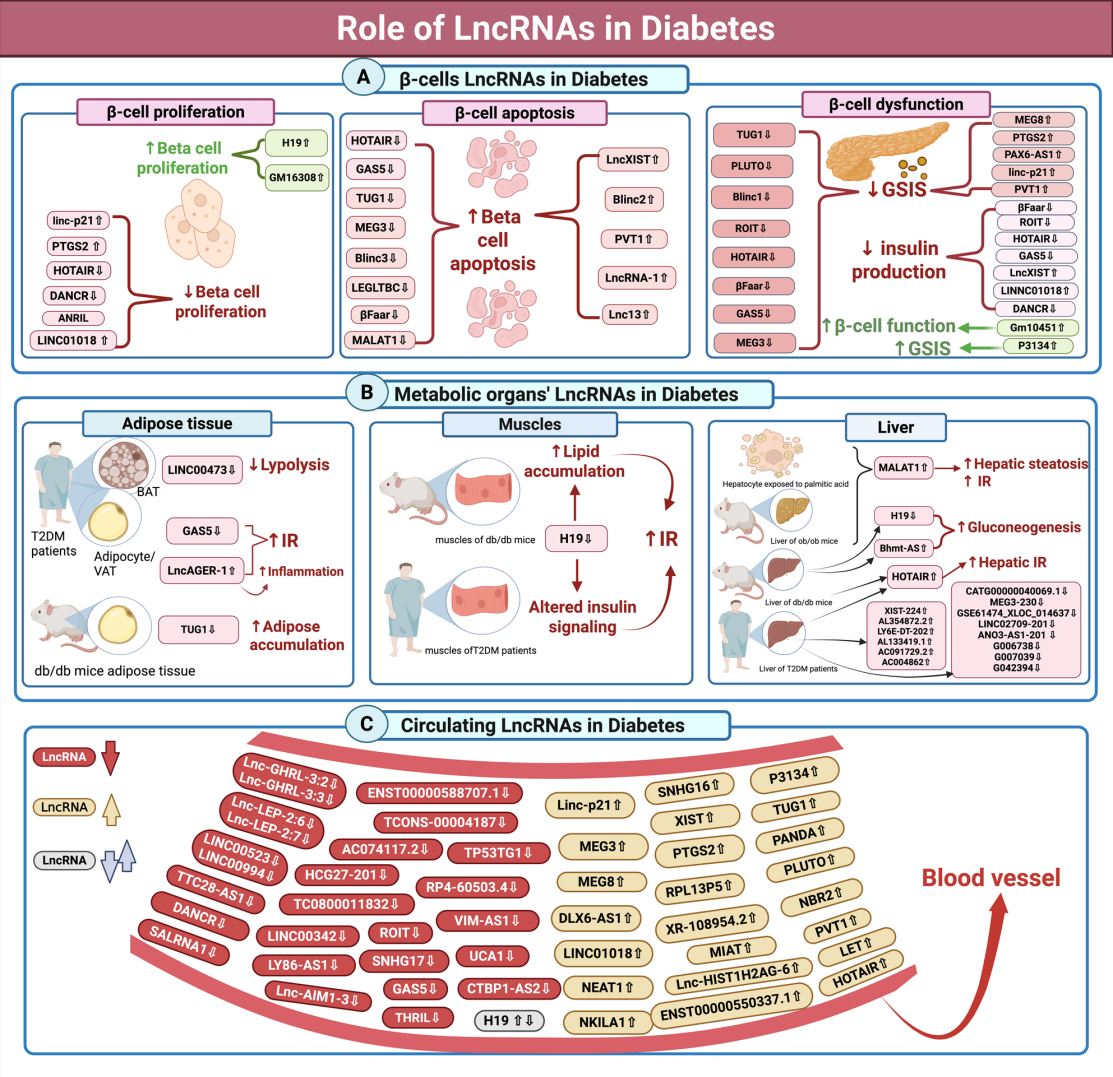
The roles of various lncRNAs in diabetes. This figure illustrates the differential expression and functional contributions of lncRNAs in the progression of diabetes, including those derived from (**A**) Pancreatic β-cells, (**B**) Metabolic organs, and (**C**) Circulating lncRNAs. *GSIS: glucose-stimulated insulin secretion; IR: insulin resistance.* This figure was created in https://BioRender.com

Among the diverse cellular communicators implicated in diabetes, lncRNAs have recently gained significant attention as novel regulatory molecules. Emerging evidence suggests that lncRNAs can influence the key mechanisms underlying diabetes, including insulin signaling, β-cell function, inflammation, lipid metabolism, and epigenetic regulation [49–54]. Various roles of lncRNAs in DM are summarized in Table 1 and illustrated in Fig. 2.

**Table 1:** Role of various LncRNAs in diabetes mellitus

LncRNA	Levels	Model	Effect of dysregulated levels on diabetes	Mechanism of lncRNAs	Clinical importance	Ref.
Gm16308 (Lnc03)	↑↑	MIN 6 cell, mouse β-cell treated with prolactin and mouse β-cell during pregnancy	↑ β-cell proliferation	↑β-cell proliferation either by the stimulation of prolactin via STAT5 pathway or in absence of prolactin	May act as a Therapeutic target in DM	[55]
DANCR	↓↓	Blood of GDM patients	Inhibit β-cell proliferation and insulin synthesis	Sponge miR-33a-5, reversing its inhibitory effect on β-cell proliferation and insulin secretion	------------	[56]
H19	↑↑	Islets of extreme obese db/db mice models	↑β-cell proliferation	Sponges miR-let-7 and activates the PI3K/AKT pathway	May act as Therapeutic target for T2DM	[57]
	↓↓	Liver of db/db mice	-↑ Hepatic gluconeogenesis -Impair insulin signaling	Regulate gluconeogenic genes by affecting FOXO1 nuclear localization ↑IR, p-IR and p- AKT	Potential therapeutic target to regulate glucose metabolism and improving insulin sensitivity in T2DM	[58, 59]
	↓↓	Muscles of T2DM patients and rodents with insulin resistance	Impair insulin signaling and reduced glucose absorption	Sponge let-7, enhancing its target genes, such as Insr and Lpl	-----------	[60]
	↓↓	Skeletal muscle of db/db mice	Impaired glucose tolerance and ↑insulin resistance	Interacted with hnRNPA1, ↑ skeletal muscle fatty acid oxidation-related genes	Potential therapeutic target for insulin resistance in T2DM	[61]
TUG1	↓↓	NOD mice islets, MIN6 and pancreatic β-cell	↑β-cell apoptosis, ↓insulin production and disrupt GSIS	- ↓Caspases 3, 7 and 12, - ↑Ins1 and Ins2 mRNA	-----	[62]
	↓↓	Islets of HFD mice induced GDM	↑ Insulin resistance in GDM mice	Activates the SREBP-2/ERK pathway via sponging miR-328-3p	May act as therapeutic target for GDM	[63]
	↓↓	Adipose tissue of diabetic mice	↑Body weight and adipose accumulation	Activates SIRT1/AMPK/ACC signaling pathway	-----------	[50]
lncAGER-1	↑↑	visceral adipose tissue of obese and T2DM patients	↑Inflammation ↑Insulin resistance	Modulates AGER protein	-----------	[49]
LncXIST	↑↑	Islet β-cell of T2DM patients and rats	↑ β-cell apoptosis and disrupt insulin production	Regulates miR-130a-3p/ALK2 pathway	May act as Therapeutic target in DM	[64]
MEG3	↓↓	Islets of T1DM and T2DM mice and T2DM donors	↑ β-cell apoptosis and impair insulin production and secretion	↑Ins2, Pdx1, and Mafa ↓Bax and caspase-3	May act as Therapeutic target in DM	[65–67]
Blinc 2	↑↑	Pancreatic islets of T2DM mice and MIN 6 cells	↑ β-cell apoptosis	Glucolipotoxicity-mediated β-cell loss	May act as Therapeutic target in DM	[68]
Blinc 3	↓↓	Pancreatic islets of T2DM mice, MIN 6 cells and T2DM donor islets				[68]
LEGLTBC	↓↓	INS-1 cells treated with HG/PA		Induce SIRT1 via Sponging miR-34a	------	[69]
PVT1	↑↑	INS-1 cells treated with STZ		↑STZ-mediated oxidative stress and apoptosis of β-cell ↓Decrease insulin secretion via regulating miR-181a-5p	May act as promising diagnostic and therapeutic target for DM	[70]
	↑↑	Circulation of prediabetic and T2DM patients	↑ Progressive inflammation in T2DM	Via pathways -PVT1/miR-214/NF-κB	They may act as biomarkers for T2DM	[71]
lncRNA-1	↑↑	MIN 6 cells treated with cytokines and islets of T1DM mice	↑ β-cell apoptosis	↑Translocation of NF-kB to the nucleus	-----	[72]
Lnc13	↑↑	Human pancreatic β-cell infected with virus CVB5	↑ β-cell inflammation and apoptosis	Induce stability of STAT1 mRNA/proinflammatory pathway	Diagnostic and therapeutic target for T1DM	[73]
βFaar	↓↓	Islets of obese (HFD), db/db mice and palmitate treated human islets	↑ β-cell apoptosis	↓ Caspase-3 and BAX and↑procaspase-3 and BCL-2 -Facilitating degradation of TRAF3IP2.	Therapeutic target for DM associated with obesity	[74]
			↓ Insulin transcription and secretion	-Sponging miR-138-5p, ↑expression of islet-specific genes; Ins2, NeuroD1, and Creb1		
PLUTO	↓↓	Islets of T2DM donors, MIN6 cells, EndoC-bH3 cells	↑ β-cell dysfunction (GSIS impairment)	Regulate expression of pdx1	--------	[52]
Blinc 1	-------	Blinc 1Knockdown in MIN 6 cells	↑β-cell dysfunction (impair insulin secretion)	↑Nkx2.2, Pax6, and/or MafB expression	May act as Therapeutic target in DM	[51]
Pax6os1/PAX6-AS1	↑↑	Islets of HFD mice and T2DM patients	β-cell dysfunction (impair insulin secretion)	↓ β-cell signature genes as insulin	May act as Therapeutic target in DM	[54]
MALAT-1	↑↑	Sera of diabetic patients who smokes and MIN 6 cells exposed to CSE	β-cell dysfunction (impair insulin secretion)	MALAT-1/miR-17/TXNIP pathway	Understanding how cigarette smoke impairs β-cell function via MALAT-1	[53]
	↑↑	Islets oF NOD mice and MIN 6 cells exposed to IL-1β	β-cell dysfunction (impair insulin secretion)	↓pdx1	May act as Therapeutic target in T1DM	[75]
	↓↓	Islets of db/db mice and palmitate-treated MIN 6 cells	β-cell dysfunction and β-cell apoptosis	MALAT/Ptbp1/PKM2 pathway	May act as Therapeutic target in T2DM	[76]
	↑↑	Hepatocytes exposed to palmitate and livers of ob/ob mice.	↑Hepatic steatosis and insulin resistance	↑Nuclear SREBP-1 C protein stability	May act as therapeutic target for obesity and T2DM	[77]
	↑↑	Circulation of T2DM patients	↑ Insulin resistance	Sponge miR-382-3P, ↑ resistin	May act as Therapeutic target in T2DM	[78]
	↑↑	Circulation of prediabetic and T2DM patients	↑ Progressive inflammation in T2DM	-Via pathway MALAT1/miR-9/NF-κB	Potential biomarkers for T2DM	[71]
ROIT	↓↓	Islets of T2DM animal models and serum of T2DM patients	↓Insulin expression and poor GSIS	Blocks Nkx6.1 promotor methylation	May act as Therapeutic target in DM	[79]
Gm10451	↑↑	Mouse iPSCs-derived β-like cells invitro	Induce Insulin+/Nkx6.1 + β-like cell differentiation	Suppress miR-228-3p, ↑PTIP and ↑mature beta cell markers as insulin, Mafa, and Nkx6.1		[80]
GAS5	↓↓	Diabetic human serum and islets of db/db mice	↓Insulin synthesis and production	↑Expression of insulin gene, Pdx1 and Mafa.	May act as biomarker for prediction and diagnosis of DM	[81]
	↓↓	Serum of T2DM patients	↓GSIS and insulin content	-Sponges miR-29a-3p, miR-96-3p, and miR-208a-3p, -↑INSR, IRS-1 and PIK3R1	Could serve as a promising therapeutic target for T2DM	[82]
	↓↓	Dexamethasone treated human β-cells line EndoC-bH1	↓Insulin secretion and ↑ β-cell apoptosis	↑ Pdx1 and Nkx6.1, and SYT13 ↓SGK1 ↓Glucocorticoid receptor expression	Therapeutic target to treat glucocorticoid-induced β-cell dysfunction and diabetes	[83]
	↑↑	Islets from T2DM donors and GK rat model of diabetes	Compensatory mechanism to hinder β-cell dysfunction			[83]
	↓↓	Adipocyte of T2DM patients	↓Glucose uptake and insulin signaling in adipocytes	Regulates IR transcription	Potential therapeutic target for T2DM therapy	[84]
p3134	↑↑	Blood of T2DM patients	Maintain β-cell mass and stimulate insulin synthesis and secretion	-↑ Pdx-1, Mafa, GLUT2 and Tcf7l2 in β-cells -↑GSIS via positive modulation of PI3K/AKT/mTOR	Potential biomarker for T2DM	[85]
linc-p21	↑↑	Serum of T2DM patients	↓GSIS and β-cell proliferation	Enhance NR3C2 via sponging miR-766-3p	Potential diagnostic biomarker for T2DM	[86]
HOTAIR	↓↓	Pancreatic tissues of db/db mice	-↓Insulin secretion and synthesis -↓ β cells proliferation and enhance pancreatic β cells apoptosis	-↑ Mafa, Pdx1, and NeuroD1 -↑CyclinD1,2 and 3	May act as Therapeutic target in DM	[87]
	↑↑	Liver tissues of patients with T2DM, HFD mice and db/db mice	↑Hepatic insulin resistance	↓SIRT1 expression and the AKT/GSK pathway	---------	[88]
	↑↑	Blood stream of T2DM and prediabetics	Associated with insulin resistance	---------	Possible biomarker for diagnosis and prognosis of T2DM	[89]
PTGS2	↑↑	Serum of T2DM patients, HG treated INS-1 cells	β cells dysfunction “suppressed the growth and insulin release”	Regulates miR-146a-5p and RBP4	May act as diagnostic biomarker for T2DM	[90]
Bhmt-AS	↑↑	Liver of fasting and db/db mice	↑Hepatic glucogenesis	Bhmt-AS regulates Bhmt and gluconeogenic genes such as Pepck and G6pase.	May act as Therapeutic target in T2DM	[91]
RPL13P5	↑↑	Peripheral blood of GDM patients	↑ Insulin resistance	Via involvement in PI3K- AKT signaling system, insulin signal pathway, and TSC2 gene	Potential diagnostic and prognostic biomarker for GDM	[92]
DLX6-AS1	↑↑	Serum of GDM patients	↓ β cells Proliferation and ↑its apoptosis in the GDM cell model	Sponge miR-497-5p.	Potential diagnostic and Therapeutic biomarker for GDM	[93]
LINC01018	↑↑	Plasma of T2DM patients and HG-pancreatic β-cell	↓β-cell proliferation ↓Insulin secretion ↑ Cell dedifferentiation	Sponges miR-499a-5p	Potential diagnostic biomarker for T2DM	[94]
MEG8	↑↑	Plasma and blood of GDM patients	↓Insulin secretion and β-cell viability	Targets miR-296-3p	Potential biomarker for diagnosis and therapeutic target for GDM	[95, 96]
SNHG16	↑↑	PBMCs of T2DM patients, HG treated THP-1 cells and diabetic mice	Modulates inflammatory process in diabetes	Modulate NF-κB via sponging miR-212-3p	Potential diagnostic, prognostic biomarker, and therapeutic target for diabetes	[97, 98]

*ACC* Acetyl-CoA Carboxylase, *AGER* Advanced Glycosylation End Product–Specific Receptor, *AKT* Serine/Threonine-Specific Protein Kinase, *ALK2* Activin Receptor–Like Kinase 2, *AMPK* AMP-Activated Protein Kinase, *BCL-2* B-Cell Lymphoma 2, *Bax* BCL-2-Associated X Protein, *Bhmt* Betaine-Homocysteine S-Methyltransferase, *CSE* Cigarette Smoke Extract, *Creb1* cAMP Response Element–Binding Protein 1, *CVB5* Coxsackievirus B Group 5, *db/db mice* Diabetic Mice, *DM* Diabetes Mellitus, *EndoC-βH3 cells* Endocrine Human β-Cell Line 3,*ERK* Extracellular Signal-Regulated Kinase, *FOXO1* Forkhead Box Protein O1, *G6Pase* Glucose-6-Phosphatase, *GDM* Gestational Diabetes Mellitus, *GK* Goto-Kakizaki, *GSK* Glycogen Synthase Kinase, *GLUT2* Glucose Transporter 2,*GSIS* Glucose-Stimulated Insulin Secretion, *HG* High Glucose, *HFD* High-Fat Diet, *iPSCs* Induced Pluripotent Stem Cells, *IL-1β* Interleukin-1 Beta, *INS-1 cells* Insulinoma-1 Cells, *Ins1* Insulin 1, *Ins2* Insulin 2, *Insr* Insulin Receptor, *IR* Insulin Receptor, *let-7* Lethal-7, *Lpl* Lipoprotein Lipase, *Mafa* Musculoaponeurotic Fibrosarcoma Oncogene Homolog A, *MafB* Musculoaponeurotic Fibrosarcoma Oncogene Homolog B, *MIN6 cells* Mouse Insulinoma Cell Line, *mRNA* Messenger Ribonucleic Acid, *mTOR* Mechanistic Target of Rapamycin, *NF-κB* Nuclear Factor Kappa-Light-Chain-Enhancer of Activated B Cells, *Nkx2.2* NK2 Homeobox 2, *Nkx6.1* NK6 Homeobox 1, *NOD* Non-Obese Diabetic, *ob/ob* Obese Mouse, *PA* Palmitic Acid, *PBMCs* Peripheral Blood Mononuclear Cells, *Pax6* Paired Box 6, *Pdx1* Pancreatic and Duodenal Homeobox 1, *Pepck* Phosphoenolpyruvate Carboxykinase, *PI3K* Phosphatidylinositol 3-Kinase, *p-AKT* Phosphorylated AKT, *p-IR* Phosphorylated Insulin Receptor, *PKM2* Pyruvate Kinase Isozyme M2, *PTIP* Pax Transactivation Domain-Interacting Protein, *Ptbp1* Polypyrimidine Tract-Binding Protein 1, *RBP4* Retinol Binding Protein 4, *SGK1* Serum and Glucocorticoid-Regulated Kinase 1, *SIRT1* Sirtuin 1, *SREBP* Sterol Regulatory Element-Binding Protein, *STAT1* Signal Transducer and Activator of Transcription 1,*STAT5* Signal Transducer and Activator of Transcription 5,*STZ* Streptozotocin, *SYT13* Synaptotagmin 13, *THP-1 cells* Human Monocytic Cell Line, *T2DM* Type 2 Diabetes Mellitus, *TSC2* Tuberous Sclerosis Complex 2, *TRAF3IP2* TNF Receptor-Associated Factor 3 Interacting Protein 2, *TXNIP* Thioredoxin-Interacting Protein

#### The role of pancreatic β-cell LncRNAs in diabetes pathogenesis

The contribution of lncRNAs to pancreatic β-cell biology has emerged as a significant area of research interest in both type 1 diabetes (T1DM) and T2DM. Their expression is highly dynamic and responsive to metabolic and inflammatory stimuli, making them important regulators of β-cell homeostasis [52, 68, 72, 83]. β-Cell homeostasis encompasses the integrated regulation of β-cell proliferation, apoptosis, function, and identity [99]. The functional trends of β-cell lncRNAs are summarized in Table 2 and further illustrated in Fig. 2A.

**Table 2:** Functional trends of various β-cell LncRNAs

Category/Trend	lncRNA	Functional role	Ref.
Pro-proliferative	Gm16308 (Lnc03)	STAT5-mediated β-cell proliferation	[55]
	DANCR	Sponges miR-33a-5p →↑ β-cell proliferation	[56]
	H19	Activates PI3K/AKT → compensatory β-cell expansion	[57]
	KCNQ1OT1	sponges miR-15b-5p → releasing Cyclin D1/D2 → driving G1/S cell-cycle progression	[100]
	HOTAIR	Sustaining cyclin expression required for G1/S cell-cycle progression	[87]
Anti-proliferative	ANRIL	Senescence-associated lncRNA at the CDKN2A/B locus	[101, 102]
	PTGS2	Sponges miR-146a-5p → ↑ RBP4 → ↓ β-cell proliferation	[90]
	linc-p21	Sponges miR-766-3p → ↑ NR3C2 → ↓ β-cell proliferation	[86]
	LINC01018	Sponges miR-499a-5p → ↓ β-cell proliferation	[94]
Pro-apoptotic	Blinc2	↑ β-cell apoptosis, reducing cell viability	[68]
	PVT1	Sponges miR-181a-5p → ↑ oxidative/apoptotic signaling → ↑ β-cell apoptosis	[70]
	LncXIST	Sponges miR-130a-3p → induces iron overload and oxidative stress → β-cell apoptosis	[64]
	lncRNA-1	Activates NF-κB pathway → ↑ inflammatory apoptosis → β-cell loss	[72]
	Lnc13	Stabilizes STAT1 mRNA → ↑ inflammatory gene expression → ↑ cytokine-induced β-cell apoptosis	[73]
Anti-apoptotic	Blinc3	Protect against β-cell apoptosis.	[68]
	LEGLTBC	Safeguard β-cells against glucolipotoxicity/oxidative stress-induced apoptosis by sponging miR-34a	[69]
	TUG1	Maintains ER homeostasis → restrains caspase activity → apoptosis resistance	[62]
	MEG3	keeps Bax and caspase-3 low◊ apoptosis resistance	[65]
	βFaar	Sponges miR-138-5p → ↓ TRAF3IP2 → ↓ NF-κB signaling → ↓ β-cell apoptosis	[74]
	NONHSAG011351	Upregulates ERBB3, protecting β-cells from cytokine-induced apoptosis	[103]
	P3134	Reducing caspase-3 activation and protecting β-cells from stress-induced apoptosis	[85]
Increase insulin secretion/maintain identity	Blinc1/HI-LNC15	Maintains Nkx2.2 activity to support β-cell maturation, insulin gene expression, and proper GSIS	[51]
	PLUTO	Promotes insulin secretion by ↑ pdx1 expression	[52]
	Gm10451	Promotes insulin secretion by sponging miR-338-3p to increase PTIP expression and promote β-cell maturation	[80]
	ROIT	Promote insulin secretion by preserving Nkx6.1 expression	[79]
	DANCR	Sponges miR-33a-5p → ↑ Ins1/Ins2 → ↑ insulin secretion and preserve β-cell identity	[56]
	TUG1	Maintains β-cell function by supporting Ins1/Ins2 expression and GSIS	[62]
	MEG3	Enhance Mafa → ↑ insulin secretion and preserved β-cell identity	[66]
	βFaar	Sponges miR-138-5p to preserve insulin synthesis and GSIS, thereby supporting β-cell functional identity during metabolic stress	[74]
	P3134	↑ Ins1/Ins2 →↑ insulin synthesis and enhances GSIS	[85]
	HOTAIR	↑ Ins1/Ins2 expression → ↑ GSIS	[87]
Decrease insulin secretion/impair identity	Pax6os1/ PAX6-AS1	Suppresses insulin secretion by reducing pax6 levels and impairing β-cell identity	[54]
	MEG8	Inhibit insulin secretion and β-cell viability via sponging miR-296-3p	[95]
	PTGS2	Sponges miR-146a-5p → ↑RBP4 → impairs GSIS and weakens β-cell functional identity	[90]
	linc-p21	Sponges miR-766-3p → upregulates NR3C2 → impairs insulin secretion and weakens β-cell identity	[86]
	LINC01018	Sponges miR-499a-5p → suppresses Pdx1/Mafa/Nkx6.1→ impairs insulin synthesis and β-cell identity	[94]
	PVT1	Sponges miR-181a-5p ◊↓ insulin gene expression and GSIS	[70]
	LncXIST	Reduces GSIS by promoting iron-induced β-cell dysfunction	[64]

*AKT* Serine/Threonine-Specific Protein Kinase, *Bax* BCL-2-Associated X Protein, *CDKN2A/B* Cyclin-Dependent Kinase Inhibitor 2 A and B, *ER* Endoplasmic Reticulum, *ERBB3* Erb-B2 Receptor Tyrosine Kinase 3, *GSIS* Glucose Stimulated Insulin Secretion, *Ins1* Insulin 1, *Ins2* Insulin 2, *Mafa* Musculoaponeurotic Fibrosarcoma Oncogene Homolog A, *NF-κB* Nuclear Factor Kappa-Light-Chain-Enhancer of Activated B Cells, *Nkx2.2* NK2 Homeobox 2, *Nkx6.1* NK6 Homeobox 1, *NR3C2* Nuclear Receptor Subfamily 3, Group C, Member 2, *Pax6* Paired Box 6, *pdx1* Pancreatic and Duodenal Homeobox 1, *PI3K* Phosphatidylinositol 3-Kinase, *PTIP* Pax Transactivation Domain-Interacting Protein, *RBP4* Retinol Binding Protein 4, *STAT1* Signal Transducer and Activator of Transcription 1,*STAT5* Signal Transducer and Activator of Transcription 5,*TRAF3IP2* TNF Receptor-Associated Factor 3 Interacting Protein 2

Adult pancreatic β-cells can expand to meet increased metabolic demands, such as during pregnancy and obesity [104]. This adaptive growth during pregnancy is also accompanied by altered expression of specific pregnancy-associated lncRNAs [55, 56]. Besides, several lncRNAs have also been shown to regulate β-cell proliferation in diabetes [57, 101, 102].

As for β-cell apoptosis, it is a significant component in the pathogenesis of T1DM [105], and also T2DM [106]. However, the extent to which β-cell apoptosis, dysfunction, and dedifferentiation participate in the development of T2DM is a subject of debate [107]. LncRNAs have been identified as crucial modulators of β-cell apoptosis in different diabetic settings [62–66, 68–70], including inflammatory stress [72, 73, 103].

Beyond their roles in proliferation and apoptosis, it’s important to point that lncRNAs are also key regulators of β-cell identity and insulin secretion [51, 52, 54, 79, 80, 95]. Loss of β-cell identity disrupts insulin secretion in T2DM [107]. Also, β-cell functional decline often precedes apoptosis in T1DM [108], with abnormal glucose-stimulated insulin secretion (GSIS) detectable years before diagnosis [109]. Several lncRNAs enhance β-cell function [51, 52, 79, 80], while others contribute to β-cell impairment [54, 95]. Additionally, some lncRNAs negatively impact both β-cell activity and proliferation [86, 90, 94].

Controversially, some lncRNAs have been reported to have controversial roles in diabetes pathogenesis. For example, on one hand, research findings emphasized the role of metastasis-associated lung adenocarcinoma transcript 1 (MALAT1) in regulating β-cell function via miR-17 modulation, thereby affecting insulin synthesis [53]. Additionally, MALAT1 levels were upregulated in T1DM models, including interleukin-1 beta (IL-1β)–treated mouse insulinoma 6 (MIN6) cells and diabetic mouse islets. This upregulation inhibited Pancreatic and Duodenal Homeobox 1 (Pdx1), resulting in impaired insulin secretion [75]. On the other hand, in T2DM, a recent study reported low MALAT1 expression levels in db/db mice and palmitate-treated MIN6 cells. This low expression has been linked to low β-cell function and induced β-cell apoptosis [76]. These opposing findings likely reflect fundamental differences in inflammatory and metabolic signaling between T1DM and T2DM, as well as variations in experimental models, and also highlight possible context-dependent roles of MALAT1.

Similarly, the lncRNA growth arrest-specific 5 (GAS5) expression was reduced in the islets of db/db mice [81]. GAS5 downregulation was associated with reduced GSIS [81–83]. In agreement with this, Esguerra et al. reported reduced GAS5 expression in human β-cell exposed to corticosteroids linking reduced GAS5 expression to the pathogenesis of diabetes. Importantly, the same study showed that GAS5 was upregulated in the islets from T2DM patients and the T2DM model Goto-Kakizaki (GK) rats as well as the EndoC-bH1 β-cell exposed to hyperglycemia. These contradictory findings were discussed in the light of role of GAS5 as repressor of glucocorticoid receptor transcriptional activity. In response to hyperglycemia, GAS5 is upregulated as a compensatory mechanism to further repress the glucocorticoid receptor pathway to prevent beta cell failure. However, this upregulation of GAS5 is not sufficient to prevent reduction in β-cell specific transcription factors and eventually β-cell dysfunction occurs [83]. Notably, the expression of GAS5 in response to glucotoxicity is variable depending on concentration of glucose. GAS5 expression in MIN6 cells decreased at high concentration of glucose, while, in EndoC-bH1 β-cell, higher concentration of glucose reduced the upregulated GAS5 expression compared to the lower dose [81, 83]. These controversial findings can be due to different in vitro models and different conditions of glucotoxicity, and also highlight possible context-dependent roles of GAS5.

#### The role of various metabolic tissues’ LncRNAs in diabetes pathogenesis

Specific lncRNAs, expressed in key metabolic organs, have been demonstrated to participate in the complex biological processes involved in diabetes pathogenesis [110]. Those lncRNAs are summarized in Table 1 and illustrated in Fig. 2B.

 In adipose tissue, several lncRNAs showed dysregulated expression in obesity and T2DM. For instance, long intergenic non-protein coding RNA 473 (LINC00473) was shown to be uniquely detected in human brown adipose tissue (BAT) but was lowered in the BAT of obese and T2DM patients [111]. Similarly, the levels of lncRNA TUG1 were significantly decreased in the adipose tissue of diabetic mice. However, overexpressing TUG1 significantly boosted adipose oxidation and slowed diabetes development [50]. Additionally, it was found that the coexistence of obesity and diabetes led to elevated levels of lncRNA AGER-related transcript 1 (lncAGER-1) in adipose tissue, which may promote inflammation, thereby increasing insulin resistance and T2DM risk [49].

 In skeletal muscle, lncRNAs critically regulate muscle biogenesis and insulin sensitivity. In insulin-resistant C2C12 myoblasts treated with palmitic acid, 70 lncRNAs were upregulated and 74 downregulated, many linked to insulin signaling [112]. Among these, H19 expression was markedly reduced in the muscles of T2DM individuals, db/db mice, and rodent models exhibiting insulin resistance [60, 61]. H19 downregulation disrupted insulin signaling and glucose uptake by modulating the key targets involved in fatty acid oxidation [60]. In line with that, it was reported that reduced H19 expression facilitated fat buildup and contributed to insulin resistance [61], while its presence improved muscle insulin sensitivity [113].

 In the liver, a central organ in metabolic regulation and T2DM development [114], multiple lncRNAs show differential expression in diabetic patients. Elevated lncRNAs, include XIST-224, AL354872.2, LY6E-DT-202, AL133419.1, AC091729.2, and AC004862 whereas others like CATG00000040069.1, MEG3-230, GSE61474_XLOC_014637, LINC02709-201, ANO3-AS1-201, G006738, G007039, and G042394 were found to be downregulated [115].

Specific lncRNAs have a role in hepatic gluconeogenesis in diabetes. Functionally, H19 is downregulated in diabetic mouse liver, leading to increased gluconeogenic gene expression mediated via forkhead box protein O1 (FOXO1) [58, 59]. Conversely, lncRNA betaine-homocysteine methyltransferase antisense RNA (Bhmt-AS) expression increased significantly in db/db mice the liver of db/db mice, enhancing hepatic gluconeogenesis. However, Bhmt-AS downregulation reversed the process in vivo and in vitro [91].

These outcomes demonstrate the significance of tissue-specific regulatory functions of lncRNAs in metabolic disorders, linking them directly to insulin resistance, lipid metabolism, and gluconeogenesis, positioning them as promising targets for novel diagnostic and treatment approaches for diabetes.

#### The role of Circulating LncRNAs in diabetes

Circulating lncRNAs represent potential diagnostic, prognostic and therapeutic biomarkers in diabetes due to their differential expression and their functional involvement in diabetes pathogenesis. Several circulatory lncRNAs showed altered expression in T2DM compared to healthy controls, implicating their roles in metabolic dysregulation. For instance, the lncRNA XR_108954.2 was upregulated in the peripheral blood mononuclear cells (PBMCs) of T2DM and modulates insulin secretion pathways [116]. Conversely, the lncRNAs ENST00000588707.1 and TCONS_00004187 exhibit significant downregulation in PBMCs, potentially influencing glycolipid metabolism [117]. Besides, elevated lncRNA small nucleolar RNA host gene 16 (SNHG16) levels in PBMCs correlated with inflammatory processes in diabetes [97, 98].

Distinct circulating lncRNAs exhibit differential expression between T2DM, T1DM, and prediabetes. For example, a marked difference in expression levels was seen in four lncRNAs (MSTRG.128697, MSTRG.74858, MSTRG.63013, and ENSG00000269902) between T1DM and T2DM patients [118]. ENST00000480633 and ENST00000462720 levels showed substantial differences between T2DM and prediabetic patients [119]. In addition, hox transcript antisense RNA (HOTAIR) was upregulated in T2DM and prediabetic patients and was associated with insulin resistance [89].

Current research reveals conflicting expression patterns of specific circulating lncRNAs in T2DM. For example, lncRNA ANRIL expression in PBMCs of T2DM patients shows contradictory trends. These contradictions are likely due to differences in patient characteristics, disease stage, inflammatory status, and experimental approaches. For instance, a study reported increased ANRIL in T2DM patients with pronounced inflammation and senescence activation [120], whereas another study reported reduced ANRIL in T2DM examined cohorts with lower senescence marker expression and distinct metabolic profiles [102]. Similarly, circulatory MALAT1 levels in T2DM patients vary significantly across studies, with some demonstrating an upregulation in serum and leukocytes [78, 121], while others reporting a downregulation in PBMCs [122]. These contradictions may be due to the differences in sample sources, the influence of body mass index (BMI), or the effect of metformin treatment on the lncRNA expression. Likewise, H19 displayed circulatory-level discrepancies in T2DM, where elevated concentrations correlated with poor glycemic control [123]. In contrast, other studies reported reduced concentrations in patients with T2DM [124, 125]. These contradictory levels may be due to different patients’ criteria specifically the duration of diabetes, the status of glycemic control, and the associated complications. Collectively, the conflicting findings regarding ANRIL, MALAT1, and H19 levels may suggest their multifaceted role in diabetes pathophysiology. This needs further investigation into the underlying mechanisms and external factors influencing their expression.

Furthermore, distinct alterations in circulatory lncRNA expression levels have been observed in gestational diabetes mellitus (GDM) patients. Specifically, lncRNA DANCR levels were reduced, while lncRNA ribosomal protein L13 pseudogene 5 (RPL13P5) levels were elevated [56, 92]. Additionally, increased levels of MEG8 and distal-less homeobox 6 antisense RNA 1 (DLX6-AS1) have been reported in GDM patients [93, 95, 96]. DLX6-AS1 expression correlated strongly with blood glucose levels and affected cellular growth and programmed cell death [93]. Conversely, lncRNA GAS5 was significantly decreased in GDM patients [126], mirroring similar reductions seen in T2DM [122, 127]. These results underscore the possible regulatory functions of these lncRNAs in the development of GDM.

Moreover, several other circulating lncRNAs were differentially expressed in diabetes [71, 85, 102, 116, 119, 120, 128–140]. Figure 2C summarizes various circulating lncRNAs in diabetes.

**Fig. 3:**
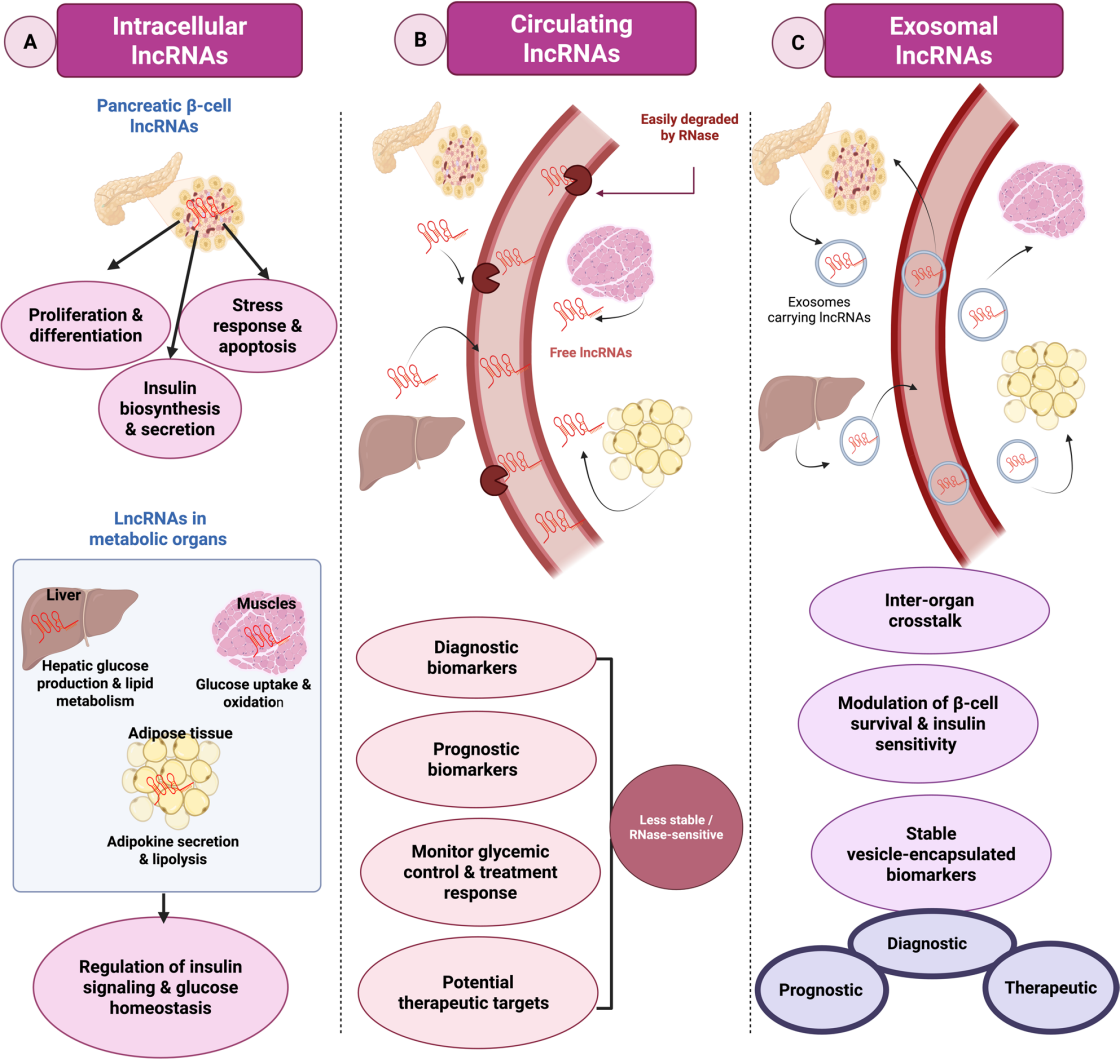
Summary of functional roles of intracellular, circulating and exosomal lncRNAs in diabetes. (**A**) Intracellular lncRNAs in pancreatic β-cells and metabolic organs, regulate insulin biosynthesis and secretion, cell survival and glucose/lipid metabolism, thereby contributing to systemic glucose homeostasis. (**B**) Tissue-derived lncRNAs released into the bloodstream as free or protein-bound molecules are relatively RNase-sensitive and may serve as diagnostic and prognostic biomarkers, indicators of glycemic control and potential therapeutic targets. (**C**) lncRNAs selectively packaged into exosomes are protected from RNase degradation, mediate inter-organ crosstalk between β-cells and metabolic tissues and provide stable vesicle-encapsulated biomarkers with diagnostic, prognostic and therapeutic potential. *LncRNA: long non coding RNA; RNase : ribonuclease.* This figure was created in https://BioRender.com

Although circulating lncRNAs contribute to intercellular communication in diabetes, their low stability limits clinical utility [141]. Interestingly, researchers discovered circulating lipid-based nanoparticle vesicles called exosomes that could protect LncRNAs from degradation [142]. A schematic summary of the functions of intracellular, circulating and exosomal lncRNAs in diabetes is shown in Fig. 3.

## Extracellular vesicles

Extracellular vesicles (EVs) are described by the International Society for Extracellular Vesicles (ISEV) as vesicles with lipid bilayers spontaneously secreted by the cells and cannot replicate [143]. Previously, EVs were thought to aid cells in eliminating their waste products [144]. However, over the past couple of decades, numerous studies have reported that EVs can transport biologically active cargoes such as nucleic acids, proteins, and lipids. These cargoes facilitate cell cross-talk and affect numerous biological pathways in the receiving cells [145, 146]. Based on EVs’ size and biogenesis, EVs are categorized into macrovesicles (MVs), apoptotic bodies (ABs), and exosomes, which are summarized in Table 3.

**Table 3:** Comparison between various types of extracellular vesicles

EVs	Size (nm)	Origin	Cargo selectivity	Types of cargo	Biological role	Ref
Exosomes	30–150	Endosomal pathway → multivesicular bodies	Highly selective (regulated sorting)	miRNAs, lncRNAs, proteins (tetraspanins: CD9/CD63/CD81), lipids, minimal DNA	Cell–cell communication, signaling, immune modulation	[147]
Microvesicles (MVs)	100–1000	Direct outward budding of plasma membrane	Moderately selective	Cytosolic proteins, receptors, integrins, RNAs, some DNA	Signaling, coagulation, inflammation responses	[148]
Apoptotic Bodies (ABs)	50–5000	Membrane blebbing during apoptosis	Non-selective, random cell fragments	Genomic DNA, mitochondrial DNA, organelles, cytoplasmic fragments, apoptotic markers	Clearance of dying cells, immune tolerance	[149]

###  Exosomes biogenesis

**Fig. 4:**
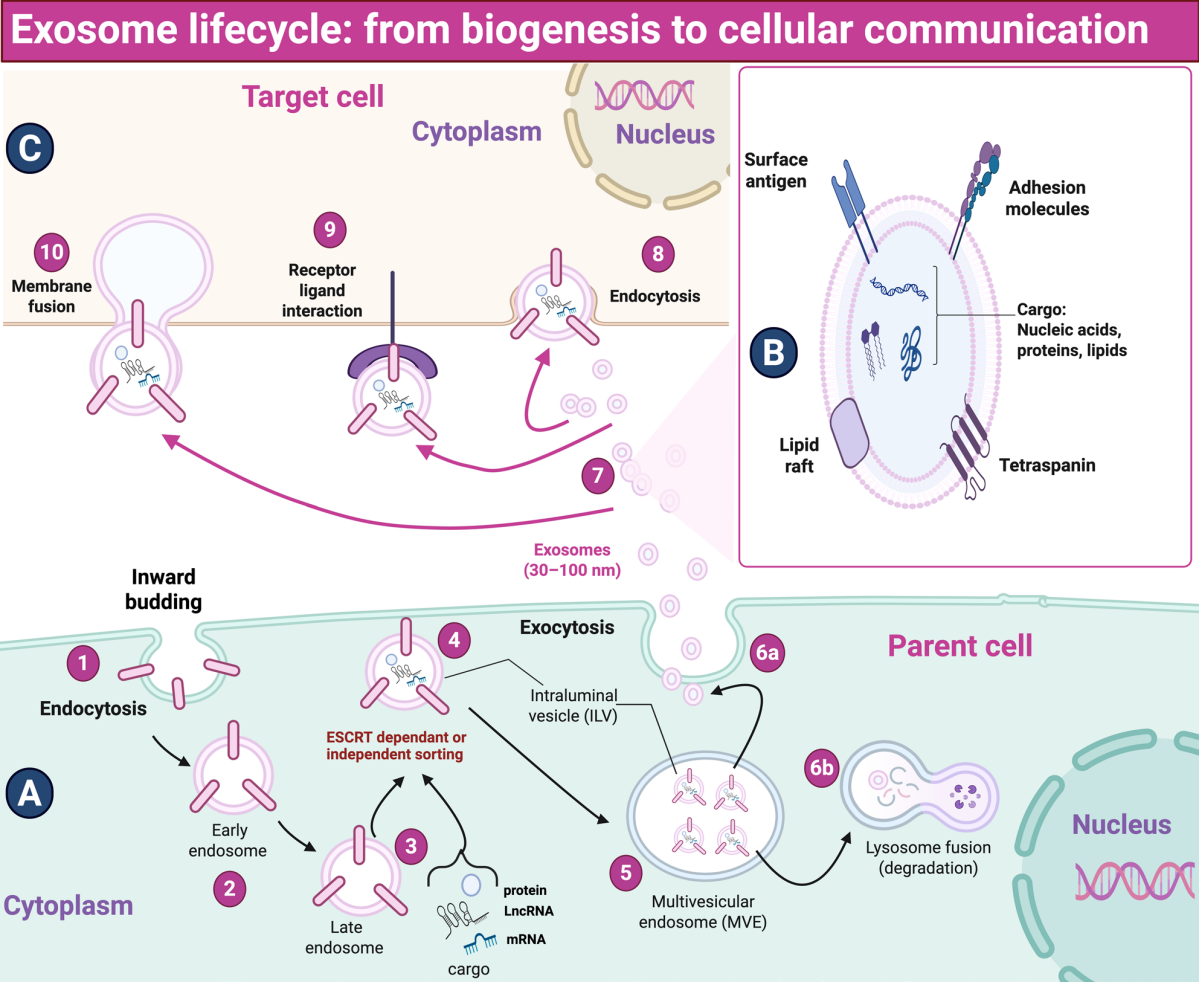
Exosome lifecycle from biogenesis to cellular communication. (**A**) Exosome biogenesis in the parent cell begins with endocytosis, forming early and then late endosomes, which are loaded with proteins, lipids, and RNAs via ESCRT-dependent or independent mechanisms to create ILVs inside MVEs. MVEs either fuse with lysosomes for degradation or with the plasma membrane to release ILVs as exosomes. (**B**) The exosome membrane is enriched with surface antigens, adhesion molecules, lipid rafts, and tetraspanins. Internally, exosomes carry a variety of bioactive molecules, such as nucleic acids (RNA/DNA), proteins, and lipids. (**C**) Mechanisms of exosome uptake in the target cell include (8) endocytosis (9) interaction between exosomes’ transmembrane proteins and signal receptors on the target cell membrane, and (10) fusion of exosome membrane with the target cell membrane, resulting in direct delivery of exosomal cargo into the cytoplasm of the target cell. *ESCRT: endosomal sorting complex required for transport; Rab27A: Ras-related protein 27 A.* This figure was created in https://BioRender.com

Exosome biogenesis begins with the inward budding of the plasma membrane to form early endosomes, which mature into late endosomes and develop intraluminal vesicles (ILVs) within multivesicular bodies (MVBs). Proteins, cytoplasmic elements, and noncoding RNAs are incorporated into ILVs during this process [150]. MVBs then fuse with the plasma membrane, releasing ILVs as exosomes [151]. Selective cargo loading ensures exosomes carry specific proteins, lipids, and RNAs, reflecting the molecular characteristics and physiological state of their cell of origin [152–154]. Exosomes’ biogenesis is illustrated in Fig. 4A and exosomal contents are illustrated in Fig. 4B.

### Exosomes as mediators of intercellular crosstalk

Exosomes are capable of transporting diverse RNA types, including lncRNAs [155]. Studies have shown that certain lncRNAs are selectively packaged into exosomes [156, 157]. This encapsulation within the exosomal membrane protects lncRNAs from RNase degradation, enhancing their stability and thereby extending their half-life in extracellular environments [158].

Exosome-mediated cell-to-cell communication is commonly generated through three mechanisms, which are illustrated in Fig. 4C and can be summarized as follows: (a) Endocytosis of donor cell-derived exosomes by the target cell, followed by the release of encapsulated bioactive cargo.

(b) Direct interaction between transmembrane proteins on exosomes and signal membrane-bound receptors of recipient cells. (c) Membrane fusion of the exosome with the recipient cell membrane, enabling the direct transfer of exosomal contents into the cytoplasm of the recipient cell [159].

### Role of exo-lncRNAs in diabetes

Exosomes are increasingly recognized as pivotal mediators in a wide range of normal and disease-related biological processes, including the progression of cancer, neurodegenerative diseases [38, 160, 161], and diabetes [22]. For example, a recent study discovered that patients with colorectal cancer (CRC) showed significantly increased levels of lncRNA NAMPT Antisense RNA 1 (NAMPT-AS) in their serum-derived exosomes relative to healthy controls. Importantly, this upregulation was associated with poorer clinical outcomes [162]. Similarly, studies in Alzheimer’s disease showed elevated plasma exosomal expression of the lncRNA 17 A and the lncRNA brain cytoplasmic RNA 200 (BC200), where lncRNA 17 A appears to contribute to neuroinflammatory processes, and BC200 may support long-term neuronal plasticity [161].

Recent investigations into diabetes pathophysiology have elucidated the critical importance of exo-lncRNAs in modulating both the initiation and development of different types of diabetes and their negative impacts [33, 123].Differential expression of some plasma exo-lncRNAs has been observed in diabetic patients compared to healthy individuals. Also, aberrant lncRNA expression was identified in exosomes isolated from the umbilical cord blood of GDM patients [163]. Additionally, a study reported that high glucose (HG)-treated tubular epithelial cell-derived exosomes exhibited upregulation of 93 lncRNAs and downregulation of 76 lncRNAs [164]. Similarly, 21 lncRNAs, including small nucleolar RNA host gene 5 (SNHG5) and clone 430049B03 RIKEN lncRNA (C430049B03Rik), exhibited variable expression in exosomes of three distinct progenitor cell lines when exposed to HG environments [165]. Moreover, exposure to proinflammatory cytokines led to notable alterations in the levels of 31 lncRNAs in human islet cell-derived exosomes [166].

#### Role of exo-lncRNAs in gestational diabetes mellitus (GDM)

It is well known that GDM arises during pregnancy mainly because of the onset of insulin insensitivity or inadequate secretion of insulin necessary to regulate normal blood glucose levels [167]. Exo-lncRNAs have emerged as essential modulators in the pathophysiology of GDM, influencing key biological processes such as insulin sensitivity, inflammatory responses, and fetal development. Throughout all trimesters, GDM patients exhibit substantially higher concentrations of placenta-derived exosomes compared to normoglycemic pregnant women, suggesting that hyperglycemia may modulate the release of placental exosomes into the systemic circulation [168]. Furthermore, recent investigations have demonstrated that exosomes isolated from the umbilical cord blood of patients with GDM, displayed differential expression of specific exo-lncRNAs, as summarized in Table 4.

**Table 4:** Differential expression of exo-lncRNAs in gestational diabetes mellitus (GDM)

Exo-lncRNA	Level of expression	Source	Ref.
GAS5	↓↓	Peripheral blood of GDM patients	​​ [126]
lnc-RXYLT1-3:2 TFDP2-7:2 COX17-2:3 ZBTB46-3:6	↑↑	Umbilical cord blood exosomes of GDM patients	​​[163]
lnc-TBC1D30-4:1 ENST00000596839.1 ZNF800-1:1 EIF4ENIF1-1:1 ATP8B3-3:1	↓↓		[163]
AC006064.4 lnc-HPS6-1:1	↑↑	Cord blood of GDM-related macrosomia patients	[169]
lnc-ZFHX3-7:1	↓↓		

Recent research has demonstrated that exo-GAS5 was significantly downregulated in the peripheral blood of GDM patients. Notably, GAS5 has been shown to interact with the proteins HECT and RLD domain containing E3 ubiquitin protein ligase 5 (HERC5) and tachykinin precursor 1 (TAC1) [126]. It suggests a potential mechanistic pathway through which GAS5 dysregulation may influence their expression or function, thereby contributing to GDM pathogenesis.

Furthermore, a prevalent complication of GDM is GDM-related macrosomia (GDM-M), which is marked by metabolic disturbances that affect infant growth and predispose offspring to obesity and diabetes later in life [170]. GDM-M is linked to distinct exo-lncRNA profiles. In particular, 372 lncRNAs exhibited differential expression in cord blood of GDM-M patients, as summarized in Table 4. Notably, elevated AC006064.4 levels in peripheral blood exosomes have emerged as a reliable predictor of GDM-M [169].

Collectively, these findings support the central role of exo-lncRNAs in the pathogenesis and the potential diagnosis of GDM and complications. However, the dynamic changes in maternal and placental metabolism throughout pregnancy are associated with trimester-specific fluctuations in exo-ncRNAs, including lncRNA profiles [171]. This biological variability poses challenges for consistent biomarker identification and clinical translation, underscoring the necessity for carefully timed sampling and standardized analytical protocols to achieve reliable diagnostic accuracy across gestational stages [171, 172].

#### Role of exo-lncRNA in T1DM

T1DM is a persistent autoimmune disorder marked by insufficient insulin production and high blood sugar levels resulting from pancreatic β-cell autoimmune destruction [173, 174]. Exosomes have a significant role in the communication between pancreatic β-cells and immune cells, a key factor in T1DM’s pathogenesis. Exo-lncRNAs can modulate inflammatory signaling or β-cell stress responses, thereby influencing immune activation and β-cell survival [175, 176]. This is concluded through comparing plasma samples from patients with T1DM and healthy participants that identified differential expression of 162 exo-lncRNAs; among these, MYL6-208, NSA2-203, NDUFS7-204, and RPL35A-208 were downregulated, suggesting a loss of protective or regulatory functions. In contrast, CCT5-212 and IK-208 were upregulated, potentially enhancing pathways that promote immune-mediated β-cell destruction [176].

Another in silico study revealed that exosomal- plasmacytoma variant translocation 1 long non-coding (PVT1) and long intergenic non-protein coding RNA 960 (LINC00960), which were derived from pancreatic islets, may contribute to the inflammatory response in T1DM through promoting immune activation or increasing β-cell sensitivity to inflammatory response, which promotes β-cell injury and death [177]. Mechanistically, exo-lncRNAs may act as ceRNAs that binds protective miRNAs allowing dysregulation of the pro-inflammatory genes’ expression. Direct mechanistic experiments on specific exosomal lncRNAs in T1DM are limited. Many mechanistic roles are inferred based on expression profiling and known RNA mechanisms. Therefore, further mechanistic investigations are required to prove the roles of exo-lncRNA in T1DM and to highlight the translational promise of exo-lncRNAs as accessible biomarkers for early detection and clinical monitoring of autoimmune diabetes.

#### Role of exo-lncRNAs in T2DM

**Fig. 5:**
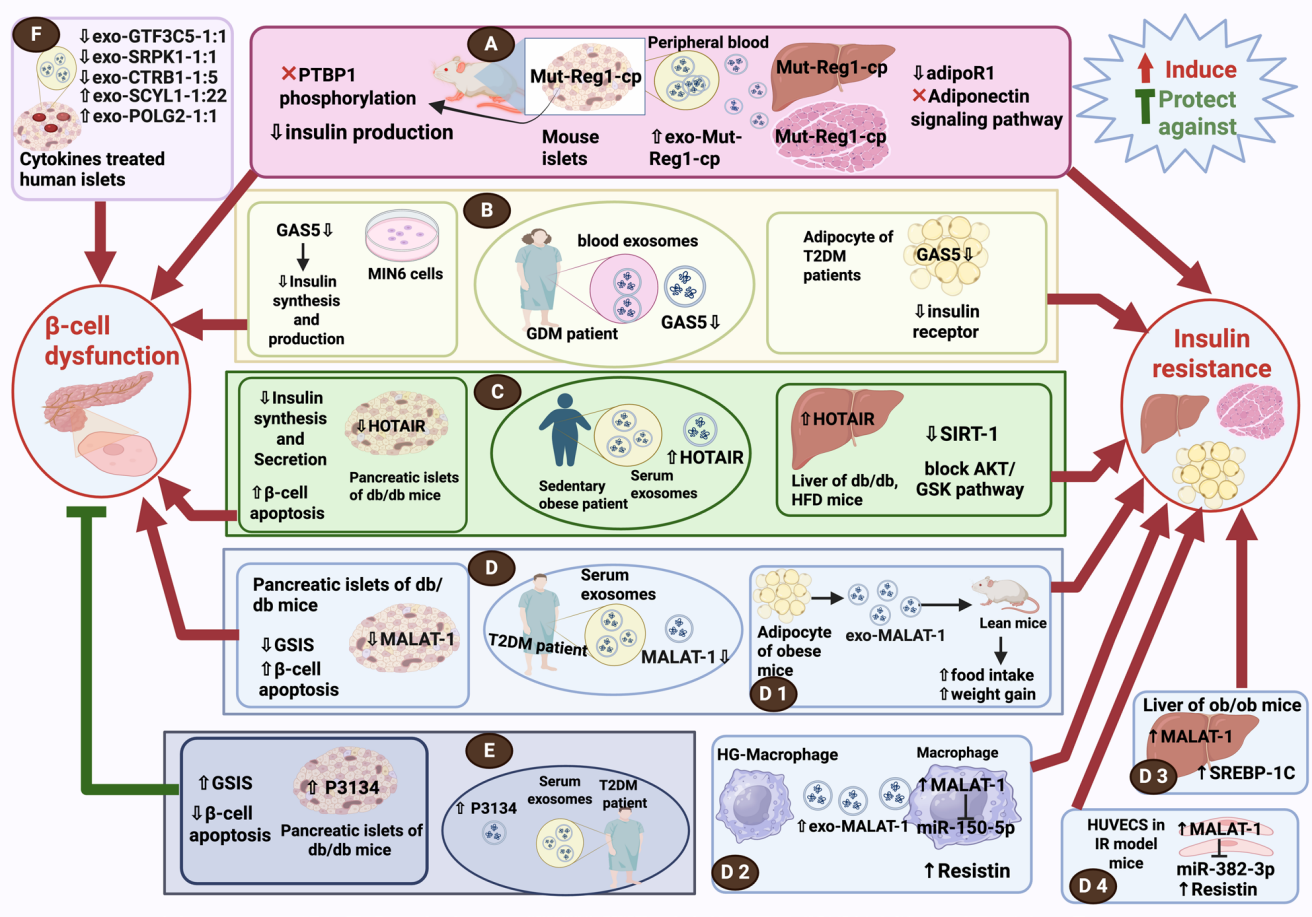
Role of exo-lncRNAs in main hallmarks of diabetes: Insulin Resistance and β-Cell Dysfunction. (**A**) Exo-Mut-Reg1-cp impairs PTBP1 phosphorylation in islets and disrupts adiponectin signaling in adipose tissue, contributing to both β-cell dysfunction and insulin resistance. (**B**) Exo-GAS5 is decreased in the blood of GDM patients. GAS5 downregulation in MIN6 cells leads to β-cell dysfunction, while its downregulation in adipocytes of T2DM patients promotes insulin resistance. (**C**) Exo-HOTAIR is increased in the serum of sedentary obese individuals. HOTAIR upregulation in the liver of db/db mice induces insulin resistance, whereas its downregulation in islets causes β-cell dysfunction. (**D**) MALAT1 is decreased in serum exosomes of T2DM patients; (**D1)** EXO-MALAT1, derived from adipocytes of obese mice, promotes insulin resistance in lean mice, (**D2**) EXO-MALAT1 secretion is increased from HG-Macrophages, promoting insulin resistance, (**D3**) MALAT1 is upregulated in the liver of ob/ob mice, promoting insulin resistance, and (**D4**) MALAT1 increased expression in HUVECs also promotes insulin resistance, contributing to systemic insulin resistance. (**E**) P3134 is upregulated in serum exosomes from T2DM patients and in islets of db/db mice, and appears to protect against β-cell dysfunction. (**F**) Deregulated expression of certain exo-lncRNAs in cytokine-treated human islets promotes β-cell dysfunction. *AdipoR1: Adiponectin Receptor 1; AKT: serine/threonine-specific protein kinase; db/db mice: diabetic mice; GDM: gestational diabetes mellitus; GSIS: glucose-stimulated insulin secretion; GSK: Glycogen Synthase Kinase; HFD: high-fat diet; HG-macrophage: high glucose–treated macrophage; HUVECS: human umbilical vein endothelial cells; IR model: insulin resistance model; MIN6 cells: mouse insulinoma cell line; Mut-Reg1-cp: mutant form of the lncRNA Regenerating islet-derived 1 alpha pseudogene; Ob/Ob mice: obese mice; PTBP1: polypyrimidine tract-binding protein; SIRT1: sirtuin 1; SREBP-1 C: Sterol Regulatory Element-Binding Protein 1 C; and T2DM: type 2 diabetes mellitus.* This figure was created in https://BioRender.com

T2DM is characterized by two main features: β-cell dysfunction, leading to reduced insulin production, and insulin resistance in peripheral tissues [178]. Exo-lncRNAs can regulate both of these hallmarks. The role of exo-lncRNAs in the hallmarks of diabetes is summarized in Fig. 5.

##### Role of exo-lncRNAs in β-cell dysfunction

Exo-lncRNAs have become key modulators of β-cell function, influencing insulin production, inflammation, and apoptosis, thereby contributing to diabetes pathogenesis and presenting potential therapeutic value [179].

Recently, it was discovered that the exosomes derived from human islets in humanized mice treated with cytokines exhibited notable downregulation of LncRNAs GTF3C5-1:1, SRPK1-1:1, and CTRB1-1:5, and upregulation of LncRNAs SCYL1-1:22 and POLG2-1:1 in comparison to the control human islets. This differential expression may reveal their involvement in β-cell stress adaptation, intercellular communication, and survival pathways in diabetic conditions [180]. Therefore, these exo-lncRNAs may serve as early indicators of islet dysfunction.

Another exo-lncRNA, p3134, was markedly increased approximately fourfold in T2DM participants compared to healthy individuals, while its levels in exosome-free serum remain unchanged. This suggests that lncRNA-p3134, which was predominantly released by pancreatic β-cells, is selectively packaged and stabilized within exosomes. Functionally, exo-p3134 enhanced GSIS by upregulating key β-cell transcriptional regulators. Upregulation of lncRNA-p3134 in MIN6 cells and db/db mice reduced β-cell apoptosis and preserved β-cell mass under both normal and glucotoxic conditions. These findings support a compensatory role for exo-p3134 in maintaining β-cell function and glucose homeostasis, potentially reversing the insulin secretory deficit in T2DM [85]. Thus, lncRNA-p3134 may serve as a therapeutic target for diabetes management.

##### Role of exo-lncRNAs in insulin resistance

Exo-lncRNAs are increasingly recognized as key players in the regulation of insulin sensitivity. These molecules are secreted in exosomes and facilitate intercellular crosstalk between metabolic tissues such as the liver, muscle, and adipose tissue [181].

Exo-lncRNAs exert their effects by regulating insulin signaling pathways, influencing glucose transporter expression, and orchestrating cellular responses to metabolic stress and inflammation. Their dynamic expression in response to obesity, hyperglycemia, and other metabolic disturbances makes exo-lncRNAs crucial regulators of systemic insulin responsiveness and potential targets for the diagnosis and treatment of insulin resistance and T2DM [88, 182–186]. Such exo-lncRNAs are mentioned in the next Sect. 3.3.3.3.

##### Prominent exo-lncRNAs acting as key regulators for hallmarks of T2DM

Three key exo-lncRNAs (HOTAIR, Mut-Reg1cp, and MALAT1) have emerged as the most prominent regulators that affect both major pathological features of T2DM; β-cell dysfunction and insulin resistance.

- **HOTAIR**

Clinically, circulating exo-HOTAIR was elevated in obese subjects with sedentary lifestyles. Similarly, a fat squeeze of the gluteal-femoral region induced the release of exo-HOTAIR into the circulation via nuclear factor kappa-light-chain-enhancer of activated B Cells (NF-κB) activation [187]. Consistently, elevated HOTAIR expression was observed in hepatic tissues of T2DM patients, high-fat diet (HFD) mice and db/db mice. Mechanistically, HOTAIR contributes to hepatic insulin resistance by suppressing sirtuin1 (SIRT1) expression and inhibiting the Serine/Threonine-Specific Protein Kinase (AKT)/glycogen synthase kinase (GSK) signaling pathway [88]. These outcomes indicate that exo-HOTAIR may also serve as a key regulator of insulin resistance in peripheral metabolic tissues.

Beyond its peripheral metabolic effects, circulating HOTAIR was also upregulated in T2DM patients [188–190]. In contrast, its expression was significantly lowered in the islets of db/db mice. Functional studies revealed that HOTAIR suppression impairs β-cell function by reducing insulin synthesis and secretion, possibly by downregulating essential transcription factors like musculoaponeurotic Fibrosarcoma Oncogene Homolog A (Mafa), Pdx1, and neurogenic differentiation 1 (NeuroD1). Moreover, HOTAIR deficiency hinders β-cell proliferation by causing cell cycle arrest and stimulates apoptosis [87].

These data may suggest a dual function of exo-HOTAIR in diabetes, raising the hypothesis that its increased systemic levels in obesity and T2DM may reflect a compensatory response to local pancreatic islet dysfunction.

- **Mut-Reg1cp**

Recent reseach reported that the mutant form of the lncRNA regenerating islet-derived 1 alpha pseudogene (Mut-Reg1cp) contributes to the T2DM development by inducing islet β cell dysfunction and impairing insulin sensitivity. Regarding insulin resistance, exosomes secreted by islets, transported Mut-Reg1cp to peripheral tissues such as the liver and skeletal muscle. The insulin resistance was induced via inhibiting adiponectin receptor 1 (AdipoR1) expression, thus interfering with adiponectin signaling [183].

However, regarding β-cell dysfunction, high levels of Mut-Reg1cp found in peripheral blood exosomes were linked to a higher risk of T2DM. Mechanistically, Mut-Reg1cp impaired insulin mRNA stability in pancreatic β-cells by inhibiting polypyrimidine tract binding protein 1 (PTBP1) phosphorylation, thereby participating in β-cell impairment in T2DM development [183].

- **MALAT1**

Exo-MALAT1 released from adipocytes of obese mice was shown to promote hyperphagia and subsequent weight gain in lean mice via stimulation of the hypothalamic mechanistic target of rapamycin (mTOR) signaling pathway [184]. Since increased adiposity contributes to systemic insulin resistance, this pathway highlights an important link between central appetite regulation and insulin resistance [185].

In parallel, MALAT1 expression was elevated in exosomes secreted from HG-treated macrophages, where it exacerbates insulin resistance via sponging miR-150-5p, resulting in elevated resistin levels [186]. Moreover, MALAT1 was upregulated in the liver of ob/ob mice, which promoted the development of liver fat accumulation and reduced insulin sensitivity by stabilizing sterol regulatory element-binding protein 1 C (SREBP-1 C) [77]. MALAT1 further promotes insulin resistance in human umbilical vein endothelial cells (HUVECs) by inhibiting resistin degradation through miR-382-3p sponging [78]. Given that endothelial insulin resistance can propagate systemic insulin resistance through multiple mechanisms [182, 191]. Collectively, these outcomes underscore the critical function of exo-MALAT1 in insulin resistance development.

Conversely, the expression levels of MALAT1 were notably reduced in serum exosomes of T2DM patients [123]. Emerging evidence suggests that, beyond its role in insulin resistance, MALAT1 is involved in β-cell impairment in diabetes. For instance, silencing MALAT1 was shown to impair GSIS and promote β-cell apoptosis in islet cells of db/db mice [76]. These data indicate that decreased MALAT1 expression may exacerbate β-cell dysfunction, highlighting its potential involvement in the development of T2DM. However, the contradictory levels of exo-MALAT1 in T2DM may highlight its compartment-specific regulation, which needs further investigation.

Section 3.3 emphasizing the role of exo-lncRNAs in pathogenesis of different types of diabetes is summarized in Table 5.

**Table 5:** Role of exo-lncRNAs in diabetes mellitus and its hallmarks

Exo-LncRNA	Type of DM/Its hallmarks	levels	Exo-source	Function	Targets	Mechanism	Potential importance	Ref.
GAS5	GDM	↓↓	Peripheral blood of GDM Chinese women (21 GDM vs. 23 healthy pregnant women)	Contributes to the development of GDM	HERC5, TAC1	Interact with HERC5 and TAC1	Novel biomarker for GDM early prediction	[126]
p3134	T2DM	↑↑	Serum samples of T2DM patients (30 Chinese T2DM patients vs. 30 controls)	Stimulate GSIS, prevent apoptosis and maintain β-cell mass	pdx1, Mafa, GLUT2, and Tcf7I2 in β-cell	Regulate insulin signaling pathway (PI3K/AKT/mTOR) signaling in islets β-cell	A better understanding of new mechanism of glucose homeostasis	[85]
GTF3C5-1:1, SRPK1-1:1, and CTRB1-1:5	β-cell dysfunction	↓↓	Human islets treated with cytokines	Regulate β-cell dysfunction apoptosis/ dysfunction	-------	Associated with loss and survival of human pancreatic islet	A potential Therapeutic biomarker for diabetes therapy	[180]
SCYL1-1:22 and POLG2-1:1		↑↑						
Mut-Reg1cp	β-cell dysfunction//T2DM		From Pancreatic islets to Peripheral blood of T2DM Chinese patients	Promote β-cell dysfunction	-Insulin mRNA - PTBP1 in pancreatic β-cells	Impairs insulin mRNA stability by inhibiting PTBP1 phosphorylation	Diagnostic biomarker for T2DM	[183]
	Insulin resistance/T2DM			Increases insulin resistance	AdipoR1in peripheral tissues	Inhibiting AdipoR1 expression, interfering with adiponectin signaling		
MALAT-1	Insulin resistance	↑↑	Adipocytes of obese mice	Increase appetite and weight gain, promoting insulin resistance	miR-181b and miR-144 in hypothalamic POMC neurons	Activation of the hypothalamic mTOR signaling pathway	-----	[184, 185]
	Insulin resistance	↑↑	HG induced macrophages	Promote insulin resistance	miR-150-5p, resistin in macrophages	MALAT-1 sponges miR-150-5p, inducing resistin, thus reduced IL-10 secretion by macrophages, thereby promoting insulin resistance	Could be therapeutic target for managing insulin resistance	[182, 186, 191]
	β-cell dysfunction/T2DM	↓↓	Serum exosomes of T2DM patients (60 Mexican T2DM patients vs. 60 controls)	Regulate GSIS and β-cell apoptosis	Ptbp1/PKM2 in Islets of db/db mice and palmitate-treated MIN 6 cells	Reduced MALAT-1 decrease GSIS and induce β-cell apoptosis via Ptbp1/PKM2 pathway	A potential biomarker for T2DM	[76, 123]

*AdipoR1* Adiponectin Receptor 1, *AKT* Serine/Threonine-Specific Protein Kinase; GDM: Gestational Diabetes Mellitus, *GLUT2* Glucose Transporter 2,*GSIS* Glucose-Stimulated Insulin Secretion, *HG* High Glucose, *HERC5* HECT and RLD Domain Containing E3 Ubiquitin Protein Ligase 5,*IL-10* Interleukin-10, *Mafa* Musculoaponeurotic Fibrosarcoma Oncogene Homolog A, *mTOR* Mechanistic Target of Rapamycin, *Pdx1* Pancreatic and Duodenal Homeobox 1, *PI3K* Phosphatidylinositol 3-Kinase; PKM2: Pyruvate Kinase Isozyme M2; POMC: Pro-opiomelanocortin; PTBP1: Polypyrimidine Tract-Binding Protein 1; T2DM: Type 2 Diabetes Mellitus; TAC1: Tachykinin Precursor 1; Tcf7l2: Transcription Factor 7 Like 2

Taken together, exo-lncRNAs are interrelated with both β-cell dysfunction and insulin resistance in T2DM through intercellular signaling. Their dysregulated levels reflect disease progression and may serve as early biomarkers. Also, targeting key exo-lncRNAs could offer new therapeutic strategies for T2DM.

### Role of exo-lncRNAs in diabetic complications

The diabetic complications are increasingly recognized as the result of vascular dysfunction driven by cellular and molecular disruptions, particularly those induced by chronic hyperglycemia and hypoxia. These complications include microvascular and macrovascular disorders, which contribute significantly to morbidity and mortality. Current treatments may slow disease progression, but they remain insufficient to fully prevent or reverse these outcomes [192].

**Fig. 6:**
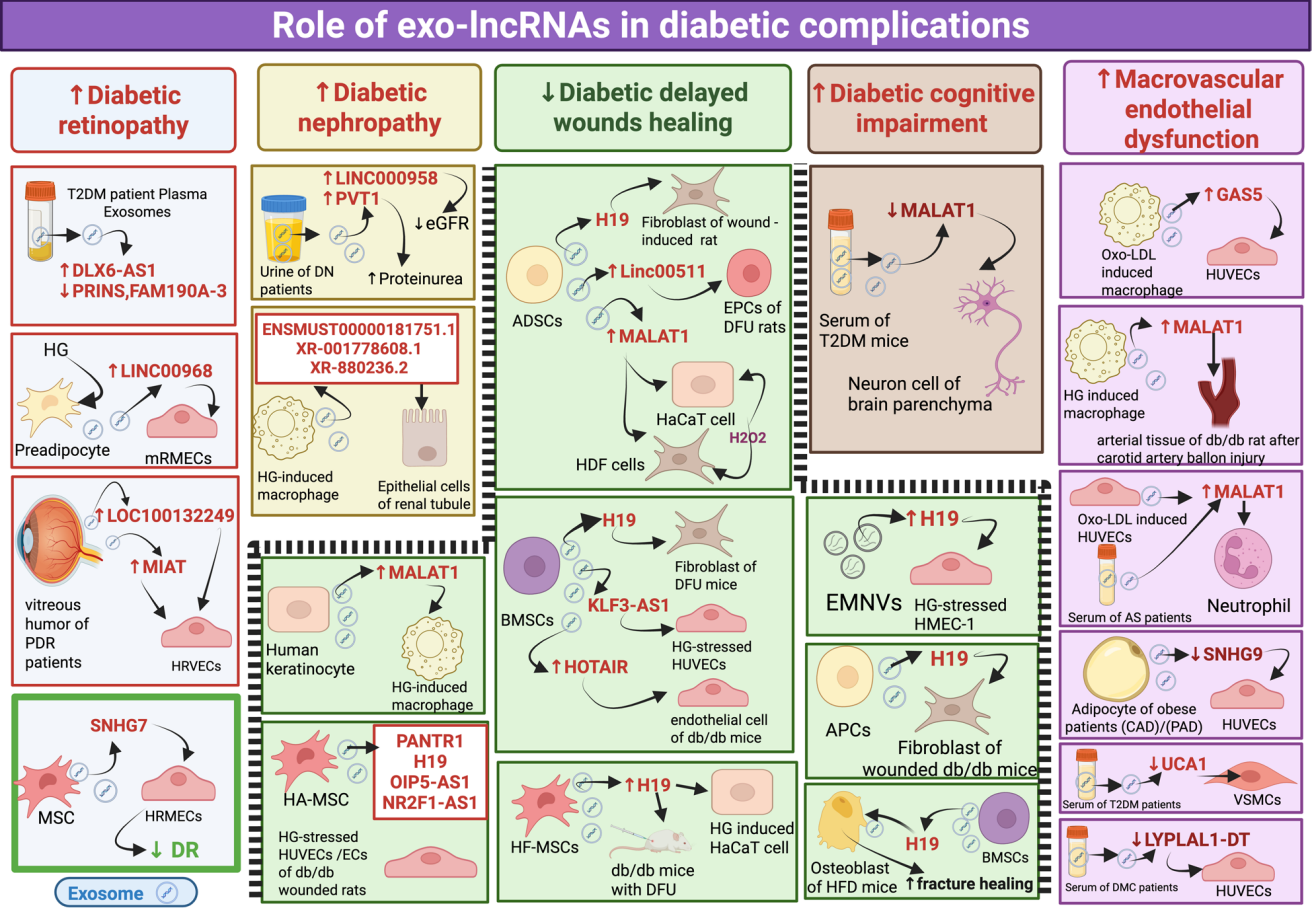
The role of exo-lncRNAs in various diabetic complications. This figure illustrates how exosomes serve as carriers of lncRNAs, facilitating intercellular communication that contributes to the progression of or protection against diabetic complications. Exo-lncRNAs are transferred from donor to recipient cells, including endothelial cells, macrophages, fibroblasts, and neurons, where they regulate key pathological processes such as inflammation, oxidative stress, angiogenesis, and tissue repair. This exo-lncRNA signaling axis is crucial in the cellular dysfunction underlying diabetic retinopathy, nephropathy, impaired wound healing, cognitive decline, and macrovascular injury. *ADSCs: Adipose-Derived Stem Cells; APCs: adipocyte progenitor cells; BMSCs: bone marrow mesenchymal stem cells; CAD: coronary artery disease; db/db: diabetic; DFU: diabetic foot ulcer; DMC: diabetic microvascular complications; DN: diabetic nephropathy; DR: diabetic retinopathy; ECs: endothelial cells; EMNVs: extracellular vesicle-mimetic nanovesicles; EPCs: endothelial progenitor cells; eGFR: estimated glomerular filtration rate; HaCaT: Human Adult Keratinocyte; HAMSC: Human Amniotic Mesenchymal Stem Cells; HDF cells: Human Dermal Fibroblasts; HF-MSCs: hair follicle mesenchymal stem cells; HMEC-1: human dermal microvascular endothelial cell-1; HRMECs: human retinal microvascular endothelial cells; HRVECs: Human Retinal Vascular Endothelial Cells; HUVECs: human umbilical vein endothelial cells; HFD: high fat diet; HG: high glucose; MSC: mesenchymal stem cell; mRMECs: mouse Retinal Microvascular Endothelial Cells; oxo-LDL: oxidized Low-Density Lipoprotein; PAD: peripheral arterial disease; PDR: proliferative diabetic retinopathy; T2DM: type 2 diabetes mellitus; and VSMCs: vascular smooth muscle cells.* This figure was created in https://BioRender.com

Recent studies have identified exosomal ncRNAs as key contributors to the development of diabetic complications, underscoring their capability as valuable diagnostic indicators and treatment targets. In particular, stem cell-derived exosomal ncRNAs, including those from mesenchymal and human urine-derived stem cells, along with exosome-targeted delivery technologies, offer promising strategies for diabetic complication therapy [193]. Among these, exo-lncRNAs attracted growing interest for their involvement in diabetes-related vascular disorders. Different roles of exo-lncRNAs in diabetic complications are summarized in Table 6; Fig. 6.

**Table 6:** Role of exo-lncRNAs in various diabetic complications

Diabetic complications	Exo-lncRNA	Level of expression	Exo-lncRNA source	Effect on diabetic complications	Potential targets	Molecular mechanism	Potential Clinical application	Ref.
Diabetic retinopathy	SNHG7	-----	Human BMSC	Slow DR by suppressing HG-induced EndMT and tube formation in HRMEC	miR-34a-5p in HRMECs	SNHG7/miR-34a-5p/XBP1 axis	Therapeutic target for the treatment of DR.	[194]
	lncRNA DLX6-AS1	↑↑	Plasma exosomes from DR patients	Its a risk factor for DR	p38–MAPK pathway	Regulating the p38–MAPK pathway inducing DR	Diagnostic biomarker for DR	[195]
	lncRNA PRINS	↓↓	Plasma exosomes from DR patients	Its reduced expression reduces SMAD 7 expression and, hinder its protective impact on the retina.	SMAD 7	TGF-β/SMAD signaling pathway		[195]
	LINC00968	↑↑	HG induced 3T3-L1 preadipocytes	↓Proliferation rates, ↑ Cell apoptosis, inflammation and oxidative stress, ultimately causing DR	miR-361–5p, TRAF3 in mRMECs	LINC00968/miR-361–5p/TRAF3 pathway	Therapeutic target for DR therapy	[196]
Proliferative diabetic retinopathy	LOC100132249	↑↑	vitreous humor of patients with PDR	Accelerate pathological angiogenesis and endothelial dysfunction in retina	miR-199a-5p, SNAI1 in HRVECs	LOC100132249/miR-199a-5p/SNAI1 axis and Wnt/β-catenin pathway		[197]
	lncRNA-MIAT	↑↑		Induce endothelial dysfunction in DR by ↑Angiogenes of HRVECs via ↑proliferation, migration, and tube formation	miR-133a-3p, MMP-X1 in HRVECs	MIAT/miR-133a-3p/MMP-X1 axis		[25]
Diabetic nephropathy	PVT1	↑↑	Urinary exosomes from DN donors	Promote renal fibrosis and dysfunction	FOXA1	Induce podocyte damage and apoptosis via downregulating FOXA1, inducing proteinuria, enhances the secretion of ECM proteins and facilitates EMT	Promising target for the diagnosis and personalized management of T2DN	[198–200]
	LINC000958	↑↑	Urinary exosomes from DN donors	Worsen renal function	------	LINC000958 negatively correlated with eGFR		[198]
	ENSMUST00000181751.1, XR_001778608.1, and XR_880236.2	↑↑	Macrophages exposed to HG	Enhancing DN	**α**-SMA, Fibronectin, E-cadherin	Promote EMT in Renal tubular epithelial cells and renal fibrosis Inducing DN via MAPK pathway	May act as therapeutic target for DN	[201]
Diabetic wounds/delayed wound healing	lncRNA H19	---	EMNVs	Promote angiogenesis in wound healing	AKT in HMEC-1	AKT activation restoration to maintain cell proliferation signals and enhance angiogenesis	Potent precision-medicine for diabetic wounds	[202]
		---	Mice ADSCs	Promote Fibroblast proliferation, migration and invasion, inducing wound healing	miR-19b in skin fibroblast	H19/miR19b/SOX9 pathway	Therapeutic target for delayed skin wound healing	[203]
		------	APCs	Promote fibroblast proliferation and macrophages recruitment, inducing wound healing	p53 and GDF15 in fibroblast	Increases fibroblast proliferation via Repressing p53 Activity and induce inflammatory macrophage recruitment via Repressing Fibroblast-Derived GDF15		[204]
		-----	HFMSCs-OE H19	Promote wound healing	NLRP3 inflammasome in HaCaT cells	↓NLRP3 inflammasome mediated pyroptosis And ↑proliferation and migration of HaCaT cells		[205]
	MALAT1	----	Human ADSC	Promote wound healing	miR-124 in skin fibroblast	MALAT1/miR-124/Wnt/β-catenin signaling pathway, enhancing cell proliferation and migration while inhibiting apoptosis.		[206]
		-------	human keratinocyte	↑macrophage phagocytosis, polarization and ↓ its apoptosis, ↑ collagen deposition,↑ angiogenesisPromoting wound healing	miR-1914-3p,,MFGE8 in macrophages	-↑ECM remodeling -↑MFGE8 ,↓ TGF-β and SMAD3 via sponging miR-1914-3p -↑VEGF, and CD31		[207]
	KLF3-AS1	-----	BMSC	Promote wound healing	miR-383, VEGFA in HUVECs	accelerating angiogenesis via KLF3-AS1/miR-383/VEGFA pathway - ↑growth, migration, and formation of tubes, while suppressing cell death in HG-stressed HUVECs		[208]
	HOTAIR	↑↑	human BMSC	Promote angiogenesis and wound healing	VEGF in HUVEC	Promote wound healing via upregulate VEGF		[209]
	PANTR1, H19, OIP5-AS1 and NR2F1-AS1	-----	Human HAMSC	Promote wound healing	VEGFA, PTEN, HIF1A, and SIRT1 in HUVECs	Induce angiogenesis proliferation, migration, and tube formation of HUVECs		[210]
DFU	lncRNA H19	---	Mice BMSC	Promote Fibroblast proliferation, migration and suppress apoptosis inducing wound healing in DFU	MiR-152–3p in skin fibroblast of DFU mice	H19/MiR-152–3p/PTEN pathway and PI3K/AKT pathway	Therapeutic target for diabetic wound healing in DFU	[211]
	linc00511	----	ADSCs	Increased angiogenesis and accelerated wound healing in DFU	Twist1,PAQR3 in EPCs of DFU rats model and patients	upregulating Twist1 levels and downregulating PAQR3 in EPCs		[212]
Delayed fracture repair	lncRNA H19	----	Normal BMSC-	Reverse the abnormal fracture healing via ↑osteogenic differentiation	miR-467 and HoxA10in osteoblast	via H19/miR-467/HoxA10 axis	May be used in treatment of diabetes’ delayed fracture repair	[213]
Diabetic cognitive impairment	MALAT1	↓↓	Serum of T2DM mice	Hinder neuronal proliferation, and induce apoptosis in brain parenchyma	miR-382-3p, BDNF	MALAT1/miR‑382‑3p/BDNF signaling pathway	Therapeutic target for cognitive impairment in diabetes	[214]
Diabetic angiopathy	lncRNA UCA1	↓↓	Serum of T2DM	Its decreased expression accelerates the development of diabetic angiopathy Due to inhibited viability and invasiveness in VSMCs under the hyperglycemia state	MiR-582-5p in VSMCs	UCA1enhances the growth and invasiveness of VSMCs under conditions of hyperglycemia by sponging miR-582-5p	Promising target for the diagnosis and therapy of T2DM and diabetic angiopathy	[215]
Diabetic endothelial /Vascular dysfunction (AS , CAD, PAD)	GAS5	↑↑	Human Macrophages treated with oxo-LDL	Induce endothelial dysfunction by increase the apoptosis of macrophagesaand ECs	↑P53, ↑Caspase 3,7,9 in HUVECs	Increase the apoptosis of macrophagesand ECs, plaques instability	Therapeutic target for the treatment of endothelial dysfunction in AS	[216]
	MALAT1	↑↑	ox-LDL-treated HUVECs and serum of AS patients	Promote atherosclerotic lesions, ROS formation and inflammation	RAC1/P38-AKT pathway in neutrophils	↑NETs via activating RAC1/P38-AKT pathway	--------	[217]
			HG stimulated Macrophages	Promote vascular disease and insulin resistance in macrophages and arterial tissues of diabetic rats following balloon injury	miR-150-5p o, resistin	MALAT-1Sponge miR-150-5p and upregulate resistin	May act as therapeutic target for vascular complications in diabetes	[186]
				Disrupting autophagy of macrophages and arterial tissues of diabetic rats following balloon injury Promoting vascular disease	miR-204-5p, LC3B in macrophages and arterial tissues of diabetic rats following balloon injury	MALAT-1 sponges miR-204-5p and upregulate LC3B		[218]
	SNHG9	↓↓	Adipocyte of obese individuals with endothelial dysfunction-associated conditions	Inducing inflammation and apoptosis in ECs	TRADD in HUVECs	Its decreased expression induces endothelial dysfunction via activation of TRADD and inhibition of NF-KB pathway	therapeutic target for the treatment of endothelial dysfunction DMC	[219]
	LYPLAL1-DT	↓↓	Serum and leukocytes of DMC patients	Promote endothelial dysfunction inducing DMC	miR-204-5p, SIRT1 in HUVEC	Its decreased expression reverses its protective effect on endothelial cell, it acts via miR-204-5p/SIRT1 pathway		[220]

*ADSCs* Adipose-Derived Stem Cells, *AKT* Serine/Threonine-Specific Protein Kinase, *APCs* Adipocyte Progenitor Cells, *AS* Atherosclerosis, *α-SMA* Alpha-Smooth Muscle Actin, *BDNF* Brain-Derived Neurotrophic Factor, *BMSC* Bone Marrow-Derived Mesenchymal Stem Cell, *CAD* Coronary Artery Disease, *CD31* Cluster of Differentiation 31, *DFU* Diabetic Foot Ulcer, *DMC* Diabetic Microvascular Complications, *DN* Diabetic Nephropathy, *DR* Diabetic Retinopathy, *ECM* Extracellular Matrix, *ECs* Endothelial Cells, *E-cadherin* Epithelial Cadherin, *EMNVs* Extracellular Vesicle-Mimetic Nanovesicles, *EMT* Epithelial-to-Mesenchymal Transition, *EndMT* Endothelial-to-Mesenchymal Transition, *EPCs* Endothelial Progenitor Cells, *eGFR* Estimated Glomerular Filtration Rate, *FOXA1* Forkhead Box A1, *GDF15* Growth Differentiation Factor 15, *HaCaT* Human Adult Keratinocyte, *HAMSCs* Human Amniotic Mesenchymal Stem Cells, *HFMSCs* Hair Follicle Mesenchymal Stem Cells, *HG* High Glucose, *HIF1A* Hypoxia-Inducible Factor 1-Alpha, *HMEC-1* Human Dermal Microvascular Endothelial Cells-1, *HoxA10* Homeobox A10, *HRMEC* Human Retinal Microvascular Endothelial Cells, *HRVECs* Human Retinal Vascular Endothelial Cells, *HUVECs* Human Umbilical Vein Endothelial Cells, *LC3B* Microtubule-Associated Protein 1 A/1B-Light Chain 3 Beta, *MFGE8* Milk Fat Globule-EGF Factor 8, *MMP-X1* Matrix Metalloproteinase-X1, *mRMECs* Mouse Retinal Microvascular Endothelial Cells, *NETs* Neutrophil Extracellular Traps, *NF-κB* Nuclear Factor Kappa B, NLRP3 inflammasome, NLR Family Pyrin Domain Containing 3 Inflammasome, *OE* Overexpression, *ox-LDL* Oxidized Low-Density Lipoprotein, *PAD* Peripheral Artery Disease, *PAQR3* Progestin and AdipoQ Receptor Family Member 3, *PDR* Proliferative Diabetic Retinopathy, *p38 MAPK* p38 Mitogen-Activated Protein Kinase, *p53* Tumor Protein p53, *PI3K/AKT* Phosphoinositide 3-Kinase/Protein Kinase B, *PTEN* Phosphatase and Tensin Homolog, *RAC1* Ras-Related C3 Botulinum Toxin Substrate 1,*ROS* Reactive Oxygen Species, *SIRT1* Sirtuin 1, *SMAD3* SMAD Family Member 3, *SMAD7* SMAD Family Member 7, *SNAI1* Snail Family Transcriptional Repressor 1,*SOX9* SRY-Box Transcription Factor 9,*T2DM* Type 2 Diabetes Mellitus, *T2DN* Type 2 Diabetic Nephropathy, *TGF-β* Transforming Growth Factor-Beta, *TRADD* Tumor Necrosis Factor Receptor Type 1-Associated Death Domain Protein, *TRAF3* TNF Receptor-Associated Factor 3, *Twist1* Twist Homolog 1, *VEGF* Vascular Endothelial Growth Factor, *VSMCs* Vascular Smooth Muscle Cells, *Wnt* Wingless/Integration 1, *XBP1* X-Box Binding Protein 1

#### Role of exo-lncRNAs in diabetic microvascular complications

Microvascular comby damage to small blood vessels, leading to significant health complications. Examples of these complications are diabetic retinopathy, nephropathy, and neuropathy [221].

##### Role of exo-lncRNAs in diabetic retinopathy

Diabetic retinopathy (DR), a frequent microvascular consequence of diabetes, stands as a leading cause of vision impairment and loss in the working-age adults worldwide. It results from chronic hyperglycemia-induced damage to the retinal microvasculature, leading to increased capillary permeability, microaneurysm formation, and progressive vision loss [222]. Current therapeutic options are limited by short half-lives, invasive administration, and long-term side effects, contributing to substantial economic and mental burdens for patients. Therefore, there is a critical necessity for innovative biomarkers to improve the diagnosis, treatment, and prevention of DR [223].

Recent research has underscored the importance of exo-lncRNAs in the development of DR, positioning them as possible molecular indicators for diagnosis and treatment intervention.For example, plasma exosomes from DR patients showed altered lncRNA expression, with exo-DLX6-AS1 upregulated and exosomal family with sequence similarity 190 member A3(FAM190A-3) and psoriasis susceptibility-related RNA gene induced by stress (PRINS) downregulated. Notably, the combination of exo-DLX6-AS1 and PRINS provided significant diagnostic utility for DR, where exo-DLX6-AS1 acted as a risk factor, while exo-PRINS and exo-FAM190A-3 served as protective factors [195].

Additionally, research indicates that mesenchymal stem cells (MSCs)-derived exosomes could transfer small nucleolar RNA host gene 7 (SNHG7) lncRNA to human retinal microvascular endothelial cells (HRMECS) and inhibit diabetic retinopathy-related processes, including endothelial-mesenchymal transition (EMT) and vascular tube development mediated by the miR-34a-5p/X-box binding protein 1 (XBP1) axis [194].

Research also demonstrates that elevated exo-lncRNAs derived from different diabetic microenvironments are fundamentally involved in promoting endothelial impairment, a key event in DR pathogenesis. Specifically, exosomal long intergenic non-protein coding RNA 968 (LINC00968) secreted from HG-induced 3T3-L13 preadipocytes promoted endothelial dysfunction through increased cell death, reduced proliferation, and inflammation. This is mediated by the LINC00968/miR-361-5p/TNF receptor-associated factor 3 (TRAF3) signaling cascade [196]. Similarly, exo-LOC100132249 and the myocardial infarction-associated transcript (MIAT), isolated from the vitreous humor of individuals with proliferative diabetic retinopathy (PDR), have been shown to induce endothelial dysfunction [25, 197]. Notably, LOC100132249 acted via the miR-199a-5p/snail family transcriptional repressor 1 (SNAI1) axis and wingless/integration 1 (Wnt)/β-catenin signaling pathway [197]. MIAT enhanced human retinal vascular endothelial cells (HRVECs) growth, motility, and tube development under HG conditions. This is mediated through the miR-133a-3p/matrix metalloproteinase-X1 (MMP-X1) axis [25].

##### Role of exo-lncRNAs in diabetic nephropathy

Diabetic nephropathy (DN) is a widespread small-vessel complication of diabetes and a major contributor to long-term renal impairment globally. It often progresses to irreversible renal dysfunction, ultimately resulting in end-stage kidney disease (ESKD), largely due to the limited availability of effective therapeutic interventions to halt its progression [224, 225].

Recent research has emphasized the essential function of exo-lncRNAs in regulating key pathological processes participating in DN, including inflammation, fibrosis, and cellular stress responses, by modulating gene expression and intercellular communication [226]. Urinary exosome-derived lncRNAs such as FLJ16779, LINC000958, and PVT1 have shown promise as non-invasive biomarkers, with distinct expression profiles in DN patients [198, 227]. Notably, LINC000958 negatively correlated with estimated glomerular filtration rate (eGFR), while PVT1 was positively associated with proteinuria and contributed to renal damage by promoting extracellular matrix (ECM) accumulation, EMT, and podocyte apoptosis [198, 199]. Mechanistic studies further revealed that silencing PVT1 can prevent podocyte injury and apoptosis by upregulating forkhead box A1 (FOXA1) expression [200]. Machine learning models further support the diagnostic value of exo-LINC000958 and PVT1 in type 2 DN [198].

Additionally, exo-lncRNAs from HG-stimulated macrophages, ENSMUST00000181751.1, XR_001778608.1, and XR_880236.2, facilitate renal fibrosis by inducing EMT in tubular epithelial cells via Mitogen-Activated Protein Kinases (MAPK) signaling [201]. These exo-lncRNAs represent promising biomarkers and therapeutic targets for DN, warranting further translational research to assess their clinical utility.

##### Role of exo-lncRNAs in impaired wound healing

Diabetic wounds are among the gravest consequences of diabetes, resulting in delayed healing and a heightened risk of limb loss due to impaired cellular functions and persistent inflammation. The underlying pathophysiology involves dysregulation of key wound-healing cells such as keratinocytes, fibroblasts, macrophages, and endothelial cells (ECs), resulting in reduced angiogenesis and deficiencies in oxygen and collagen synthesis. Despite ongoing efforts, the formulation of efficient treatment approaches to accelerate wound repair in diabetic patients remains a major challenge.

**Fig. 7:**
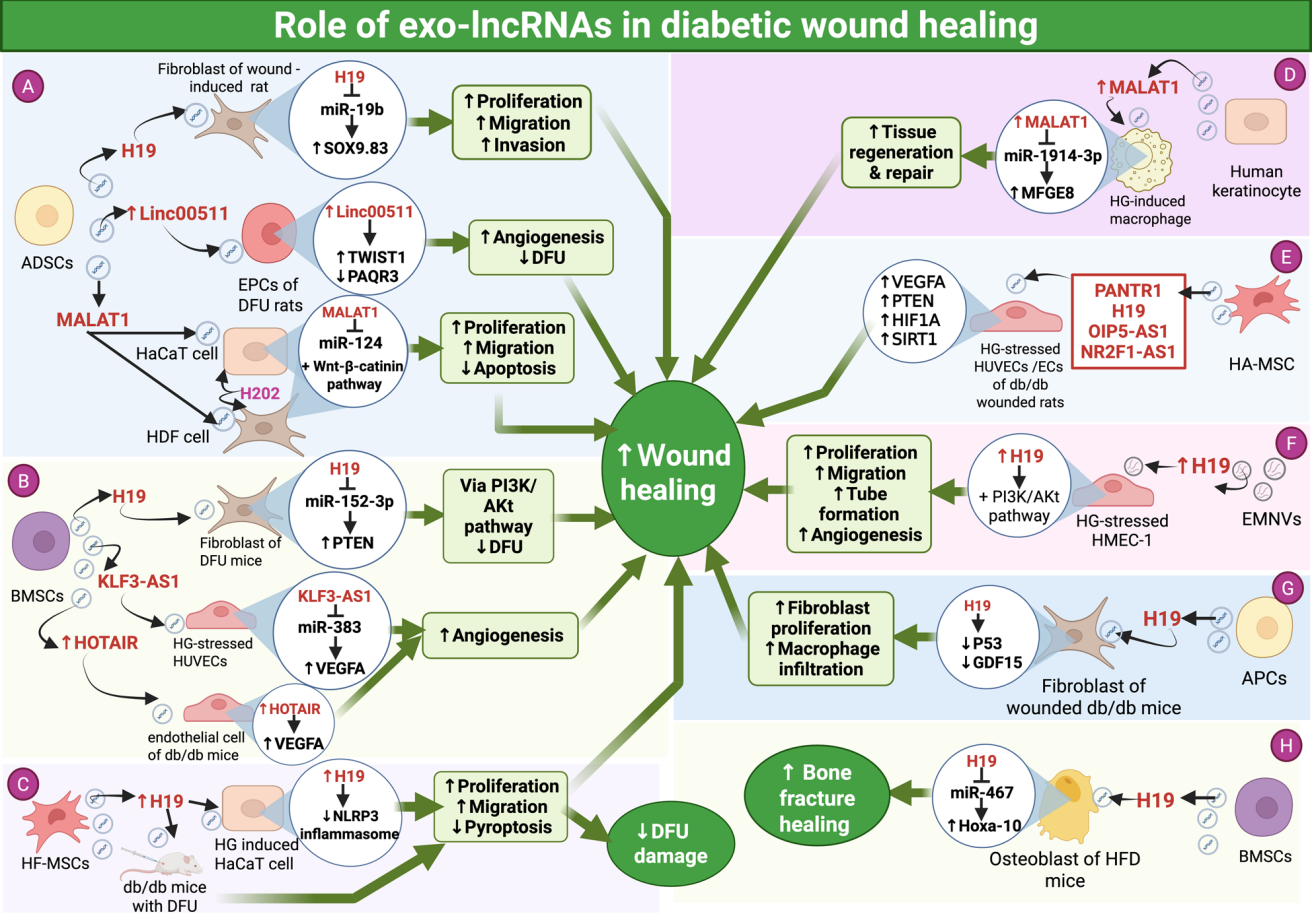
The role of exo-lncRNAs in diabetic wound healing. This figure illustrates the intercellular communication mediated by exo-lncRNAs from different stem cells to various cells in diabetic wound and fracture models, including fibroblasts, keratinocytes, endothelial cells, macrophages, and osteoblasts. These interactions promote diabetic wound healing (**A-G**) and bone fracture repair (**Hb**. The figure highlights the mechanistic pathways through which exo-lncRNAs enhance cell proliferation, migration, invasion, and angiogenesis, while inhibiting apoptosis. These processes ultimately contribute to tissue regeneration and repair in diabetic wounds and fracture models. ADSCs: Adipose-Derived Stem Cells; APCS: Adipocyte Progenitor Cells; BMSCs: Bone Marrow Mesenchymal Stem Cells; db/db: Diabetic; DFU: Diabetic Foot Ulcer; EMNVS: Extracellular Vesicle-Mimetic Nanovesicles; EPCs: Endothelial Progenitor Cells; GDF15: Growth Differentiation Factor 15;HaCaT: Human Adult Keratinocyte; HAMSCS: Human Amniotic Mesenchymal Stem Cells; HDF cells: Human Dermal Fibroblasts; HFD: High-Fat Diet; HFMSCs: Hair Follicle Mesenchymal Stem Cells; HG: High Glucose; HMEC-1: Human Dermal Microvascular Endothelial Cells-1; HoxA10: Homeobox A10; HUVECS: Human Umbilical Vein Endothelial Cells; MFGE8: Milk Fat Globule-EGF Factor 8; NLRP3 inflammasome: NLR Family Pyrin Domain-Containing 3 Inflammasome; P53: Tumor Protein p53; PAQR3: Progestin and AdipoQ Receptor Family Member 3; PI3K/AKT: Phosphoinositide3-Kinase/Protein Kinase B; PTEN: Phosphatase and Tensin Homolog; SOX9: SRY-Box Transcription Factor 9; TWIST1: Twist Homolog 1; VEGFA: Vascular Endothelial Growth Factor A; and Wnt: Wingless/Integration 1. This figure was created in https://BioRender.com

Exosomes have recently arisen as important mediators in diabetic wound repair due to their ability to modulate intercellular signalling, enhance angiogenesis, suppress inflammation, and promote collagen synthesis [202, 228, 229]. In particular, exosome-mediated delivery of lncRNAs has shown considerable potential in promoting tissue regeneration. Figure 7 summarizes the role of exo-lncRNAs in diabetic wound healing.

Among these, lncRNA H19 has been extensively studied. Its delivery via extracellular vesicle-mimetic nanovesicles (EMNVs) to human dermal microvascular endothelial cells (HMEC-1) restored H19 levels suppressed by hyperglycemia, sustaining AKT pathway activation and enhancing angiogenesis, proliferation, and migration [202]. Similarly, adipose-derived stem cell (ADSC) exosomes enriched with H19 improved wound healing in diabetic rats by targeting miR-19b and stimulating SRY-Box transcription factor 9 (SOX9). This, in turn, enhances fibroblast proliferation, migration, and invasion [203]. In another model, exosomal H19 derived from adipocyte progenitor cells (APCS) improved fibroblast proliferation and macrophage recruitment through modulation of p53 and growth differentiation factor 15 (GDF15) signalling, respectively. These mechanisms collectively improved wound healing in T2DM mouse models [204]. Furthermore, exosomes from hair follicle mesenchymal stem cells (HFMSCs) overexpressing H19 were also found to inhibit pyroptosis by suppressing the NLR family pyrin domain containing 3 (NLRP3) inflammasome, thereby promoting HaCaT keratinocyte proliferation and migration [205].

Another promising lncRNA, MALAT1, has shown beneficial effects in multiple wound-healing models. ADSC-derived exo-MALAT1 was shown to significantly enhance fibroblast growth and migration while inhibiting cell death. These effects were mediated by suppressing miR-124 and stimulating the Wnt/β-catenin pathway [206]. Furthermore, recent evidence indicated that keratinocyte-derived exosomal MALAT1 contributes to wound healing by modulating macrophage function both in vitro and in vivo. Specifically, MALAT1 acted as a molecular sponge for miR-1914-3p, thereby upregulating milk fat globule-EGF factor 8 (MFGE8) expression. This, in turn, promotes wound resolution via enhanced macrophage phagocytosis, promotes polarization, and regulates apoptotic responses. In vivo, exo-MALAT1 also facilitated collagen deposition and elevated angiogenic markers such as vascular endothelial growth factor (VEGF) and cluster of differentiation 31 (CD31), further supporting its therapeutic potential in diabetic wound management [207].

Additionally, exo-lncRNA kruppel-like factor 3 antisense RNA 1 (KLF3-AS1) derived from bone marrow mesenchymal stem cells (BMSCs) has also demonstrated therapeutic efficacy by enhancing vascular endothelial growth factor A (VEGFA) expression through miR-383 inhibition in HUVECs. This promoted endothelial proliferation, tube formation, and angiogenesis, while reducing apoptosis, which are critical for diabetic wound repair [208].

Similarly, HOTAIR-enriched exosomes from MSCs enhanced wound repair in db/db mice by upregulating VEGF in ECs, further validating the angiogenic potential of exosome-delivered lncRNAs [209]. Moreover, a recent study demonstrated that many exo-lncRNAs were correlated with angiogenesis, such as PAX8-associated non-coding transcript 1(PANTR1), H19, opa-interacting protein 5 antisense RNA 1 (OIP5-AS1), and nuclear receptor subfamily 2 group F member 1 antisense RNA 1 (NR2F1-AS1). These exo-lncRNAs were identified in human amniotic mesenchymal stem cells’ (HAMSCs) exosomes and were shown to promote diabetic wound healing in vivo via promoting angiogenesis, migration, proliferation, and tube formation in ECs of diabetic wound model rats, as well as among HG-treated HUVECs in vitro [210].

Together, these studies underscore the capability of these lncRNA-enriched exosomes as a novel class of biomolecular therapeutics for enhancing the repair of diabetic wounds through multifaceted modulation of cellular function, angiogenesis, and immune modulation.

##### Role of exo-lncRNAs in diabetic foot ulcer

Diabetic foot ulcer (DFU) is a major complication arising from poorly healing diabetic wounds on the feet. Clinical studies have demonstrated that individuals with coexisting microvascular pathologies are at significantly greater risk of developing DFUs, which contribute to diminished quality of life and increased rates of infection, gangrene, lower extremity amputation, and mortality [230–232]. Consequently, there is a critical need for effective and timely therapeutic approaches to improve healing outcomes. Emerging evidence highlights the treatment prospects of exosome-mediated delivery of lncRNAs in DFU treatment.

In a recent study, MSC-derived exosomes enriched with lncRNA H19 markedly enhanced wound healing in a DFU mouse model. These exosomes inhibited apoptosis and inflammation, while enhancing fibroblast proliferation and migration. Mechanistically, lncRNA H19 acted by sponging miR-152-3p, thereby modulating phosphatase and tensin homolog (PTEN) expression and facilitating tissue repair [211].

Furthermore, long intergenic non-protein coding RNA 511 (linc00511), delivered via ADSC-derived exosomes, showed efficacy in promoting angiogenesis and tissue regeneration in DFU. In this model, exosomes overexpressing linc00511 enhanced the function of endothelial progenitor cells (EPCS) by upregulating twist homolog 1 (Twist1) and downregulating progestin and adipoQ receptor family member 3 (PAQR3), improving neovascularisation and accelerating wound healing [212].

Together, these findings highlight the therapeutic promise of these exosome-mediated lncRNA delivery in the management of DFUs, offering a novel and targeted approach to enhance tissue repair and restore vascular integrity in diabetic patients.

##### Role of exo-lncRNAs in delayed fracture healing

Delayed bone fracture healing is a well-recognised complication of diabetes. One of the contributing factors for this delay is diabetes-induced microvascular dysfunction, which compromises the delivery of oxygen and nutrients vital for bone regeneration [233].

Beyond vascular impairment, diabetes also disrupts cellular-level mechanisms critical for bone repair. Particularly, diabetes negatively affected the secretion of BMSC-derived exosomes. These exosomes played a key role in bone regeneration by delivering osteogenic signals, including lncRNA such as H19. Under diabetic conditions, the expression of H19 was downregulated, leading to impaired osteogenic differentiation and delayed fracture healing. However, in vitro and in vivo studies confirmed that normal BMSC-derived exosomes could restore normal fracture healing via H19/miR-467/homeobox A10 (Hoxa10), laying the groundwork for future exo-lncRNAs-based fracture repair treatments [213].

##### Role of exo-lncRNAs in diabetic cognitive impairment

Diabetic cognitive impairment is increasingly attributed to microvascular complications. These complications impair cerebral perfusion, disrupt blood–brain barrier (BBB) integrity, and promote neuroinflammation, ultimately leading to white matter damage and impaired neural connectivity. This, in turn, can lead to cognitive decline in individuals with diabetes [234]. Also, extensive research has demonstrated an association between insulin resistance and cognitive decline through several interrelated mechanisms [235, 236]. In particular, insulin resistance could limit the energy supply available to brain neurons. thereby directly impairing brain function and cognitive capacity [237]. In addition, insulin dysregulation contributes to oxidative stress, neuronal inflammation, and apoptosis, further exacerbating cognitive deficits [238].

Emerging research highlights the role of exo-lncRNAs in modulating key molecular pathways involved in neuronal health and cognitive function [214]. Exo-lncRNAs’ ability to cross the BBB establishes them as strong contenders for therapeutic application in diabetes-associated cognitive impairment [161, 239]. Among these, exo-MALAT1 was shown to be crucial for neuronal survival and function in diabetes. Studies reported a substantial decline in MALAT1 expression in serum-derived exosomes from T2DM mice. MALAT1 could be transported via serum exosomes to the brain parenchyma. Its downregulation is associated with impaired neuronal proliferation and increased apoptosis. Interestingly, aerobic exercise has been found to upregulate MALAT1 expression in serum exosomes, which are subsequently taken up by hippocampal neurons. There, MALAT1 sponged miR-382-3p, resulting in increased brain-derived neurotrophic factor (BDNF) expression and improved cognitive performance in T2DM mice [214]. This suggests a novel therapeutic strategy for mitigating cognitive decline in diabetic patients through the modulation of exo-lncRNAs.

#### Role of exo-lncRNAs in macrovascular complications

Diabetic microvascular complications (DMC) are primarily driven by hyperglycemia-induced endothelial dysfunction and chronic inflammation. DMC accounts for over 50% of diabetes-related mortality. These complications substantially influence the development of atherosclerosis and subsequent heart diseases [240]. Current research highlights the involvement of lncRNAs in critical regulatory roles in the physiological and pathological processes of ECs [220]. Among these, exo-lncRNAs have gained increasing attention due to their potential as therapeutic targets for mitigating endothelial dysfunction and atherosclerosis in diabetes.

For instance, the lncRNA urothelial carcinoma-associated 1 (UCA1), was downregulated in both vascular smooth muscle cells (VSMCS) and serum exosomes of T2DM patients. UCA1 facilitates VSMC proliferation and migration under hyperglycemic conditions by targeting miR-582-5p. This may contribute to vascular repair and mitigate diabetic angiopathy. Therefore, exosomal UCA1 represents a potential biomarker and therapeutic target in T2DM-associated vascular disease [215].

Similarly, the lncRNA GAS5 was upregulated in oxidised low-density lipoprotein (ox-LDL)-treated THP-1 macrophages and their derived exosomes. These exosomes are internalised by ECs, promoting apoptosis in both macrophages and ECs. Conversely, exosomes from GAS5-knocked-down macrophages reduced apoptosis in recipient cells, underscoring the role of exosomal GAS5 in modulating intercellular communication and influencing plaque formation, stability, and atherosclerosis progression. This suggests exo-GAS5 as a promising candidate for therapy targeting endothelial dysfunction in atherosclerosis [216].

Likewise, exo-MALAT1 levels were upregulated in human umbilical vein endothelial cells (HUVECS) following ox-LDL exposure and were elevated in the serum of atherosclerosis patients. These exosomes enhance neutrophil extracellular trap (NET) formation, reactive oxygen species (ROS) accumulation, and inflammatory responses, thereby accelerating atherosclerosis progression [217]. Additionally, HG conditions markedly upregulated MALAT1 in macrophage-derived exosomes [186, 218]. In one study, MALAT-1 sponges miR-150-5p, resulting in elevated resistin expression in macrophages. This induced vascular dysfunction, contributing to atherosclerosis. Also, it was observed that exosomes produced from macrophages enhanced the expression of resistin in the arterial tissue of diabetic rats following injury to the carotid artery balloon [186]. In another study, these exo-MALAT1 also sponges miR-204-5p, resulting in elevated microtubule-associated protein 1 light chain 3 beta (LC3B) expression and impaired autophagy in macrophages, thereby promoting inflammation and vascular complications. Also, it was observed that exosomes produced from macrophages upregulated the expression of the LC3B protein in the arterial tissue of diabetic rats following injury to the carotid artery balloon [218]. These mechanistic insights collectively support the role of macrophage-derived exo-MALAT1 as a key driver in diabetes-related vascular disorders [186, 218].

Furthermore, exosomal small nucleolar RNA host gene 9 (SNHG9) derived from adipocytes was significantly reduced in obese individuals, particularly those with endothelial dysfunction-associated conditions such as coronary artery disease (CAD) and peripheral arterial disease (PAD). However, SNHG9-enriched exosomes from ADSCS were found to suppress apoptosis and inflammation in HUVECS via inhibition of tumor necrosis factor receptor type 1-associated death domain protein (TRADD), thereby preserving endothelial function. These findings suggest exo-SNHG9 as a viable therapeutic candidate for treating endothelial dysfunction in obesity-related metabolic diseases as diabetes [219].

Another lncRNA of interest is LYPLAL1 divergent transcript (LYPLAL1-DT), found to be downregulated in serum exosomes from DMC patients. In healthy individuals, LYPLAL1-DT was transferred from leukocytes to ECs via exosomes, promoting EC viability and migration under hyperglycemic stress through the ceRNA network involving the miR-204-5p/SIRT1 pathway. This mechanism enhances autophagy while inhibiting apoptosis and inflammation. Reduced exo-LYPLAL1-DT levels in DMC patients compromise these protective effects, demonstrating its suitability as a treatment target for vascular complications in diabetes [220].

These results emphasize the central role of exo-lncRNAs in modulating endothelial activity, inflammation, and vascular integrity in diabetes. This may offer opportunities for developing novel biomarkers and therapeutic strategies targeting macrovascular complications in diabetic patients.

## Therapeutic applications of exo-lncRNAs in DM and its complications

Exosomes are promising natural nanocarriers for therapeutic RNAs, owing to their biocompatibility and ability to transfer functional lncRNAs between cells. Bioengineering advances now facilitate the selective incorporation of certain lncRNAs or RNA-based oligonucleotides into exosomes, offering a targeted therapeutic platform for metabolic disorders such as DM [241–243].

### Exo-lncRNA-based oligonucleotide therapy

Engineered exosomes can transport lncRNA-modulating oligonucleotides, such as antisense oligonucleotides (ASOs) and small interfering RNAs (siRNAs), to suppress pathogenic lncRNAs or enhance protective ones. These exosome–oligonucleotide systems offer precise modulation of disease-associated lncRNA pathways [244]. Nevertheless, research in diabetic models remains limited.

### Bioengineering exosomes as LncRNA nanocarriers

Bioengineered exosomes can improve lncRNA delivery and tissue targeting. HF-MSC exosomes overexpressing lncRNA H19 enhance keratinocyte proliferation and facilitate diabetic wound healing [241]. Similarly, engineered EMNVs carrying H19 activate the PI3K/Akt pathway and enhance tissue regeneration; hydrogel-encapsulated H19-EMNVs further increase perfusion and re-epithelialization in diabetic wounds [202, 245].

Although results are encouraging, the limited number of studies highlights the need for further exploration of bioengineered exosomal lncRNAs in diabetes and its complications to support the development of future precision-medicine approaches.

## Challenges of exo-lncRNAs in clinical application

### Diagnostic challenges

The growing interest in exo-lncRNAs as diagnostic biomarkers in DM and its complications is tempered by various challenges that limit their clinical applicability. Mostly, the lack of standardized protocols for isolation and characterization of exosomes leads to variable purity, yield, and reproducibility, which compromises the consistency and comparability of results across studies [246]. Several additional concerns have also been reported, such as the typically low abundance of lncRNAs within exosomes, which complicates their detection. This requires highly sensitive and often resource-intensive technologies, especially with the potential for RNA degradation during the samples’ pre-analytical processing. These challenges, at least to some extent, might compromise the reliability of diagnostic data [247]. Another significant concern is specificity. Since exosomes are secreted by various cell types, it’s difficult to identify the exosomal cargo, such as lncRNAs, that are truly associated with the disease without strong validation [248]. In addition, combining exo-lncRNAs data with other molecular markers to improve diagnostic accuracy requires complex and still unstandardized multi-omics approaches [249, 250]. Together, these challenges highlight the need for standardized methods, improved detection technologies, and comprehensive clinical evaluation to advance the diagnostic use of exo-lncRNAs in diabetes.

### Therapeutic challenges

The therapeutic use of exo-lncRNAs in DM and its complications also shows significant promise; however, there are still several challenges to overcome, as described in recent studies. One of the biggest challenges is the difficulty of achieving efficient and tissue-specific delivery, even though exosomes have the natural ability to cross biological barriers [251]. Current targeting strategies, such as ligand modifications, are still in early stages and lack reliable in vivo precision [252]. Also, one of the main challenges is finding effective ways to load therapeutic lncRNAs into exosomes. Methods like electroporation are widely used, but they often have low efficiency and may compromise exosome integrity, which affects the consistency and quality of the final product [253, 254]. Scaling up production is another significant obstacle. It’s technically demanding to produce enough clinical-grade exosomes while ensuring each batch is consistent and meets regulatory standards [255]. There are also safety concerns to keep in mind. Even though exosomes are generally considered biocompatible, introducing engineered or foreign lncRNAs could still provoke immune responses in some cases [256]. Another important issue is the risk of off-target effects. Since lncRNAs are involved in a wide range of regulatory processes throughout the body, there’s always a possibility they could impact unintended pathways [257]. Consequently, extensive preclinical testing is essential to make sure any new therapy is both safe and effective. Ultimately, overcoming these challenges is crucial for exo-lncRNA therapies to become a practical option for treating diabetes and its associated complications.

## Future research directions for exo-lncRNAs in DM

There are several promising avenues for future research on exo-lncRNAs in diabetes that remain to be explored. Among these various future research directions, particular emphasis should be placed on validating exo-lncRNAs expression profiles that correlate with disease progression and severity, thereby enabling exo-lncRNAs to serve as non-invasive biomarkers for the early detection and monitoring of diabetes and its complications. Based on several previous reports, some exo-lncRNAs are potential candidates as biomarkers for diabetes; these can be ideally detectable in the circulation of diabetic patients and also have mechanistic links to diabetes pathology, including insulin resistance and/or β-cell dysfunction. Using these criteria, exo-p3134 [85] and exo-Mut-Reg1cp [183] can be identified as potential biomarkers for T2DM, whereas exo-GAS5 is a potential biomarker for GDM [126]. However, at present, none of them has undergone sufficient large-scale validation to be considered a clinically robust biomarker for diabetes. Thus, further investigations are indeed warranted to fully elucidate their clinical utility. It’s also important to point here that future large scale clinical studies aiming to elucidate the diagnostic/prognostic potential of exo-lncRNAs in DM should report in full detail all the statistical findings including AUC, sensitivity, specificity, etc. in order to fully elucidate/validate their potential.

Future investigations which combine exo-lncRNA data with additional biomolecular profiles, including miRNAs and circRNAs, are also needed to develop a holistic understanding of their functions in diabetes etiology. This integrative strategy may augment the predictive efficacy of exo-lncRNAs as biomarkers and therapeutic targets.

Furthermore, exo-lncRNAs, particularly those produced from stem cells, should be investigated for their therapeutic potential in diabetes and its complications. This includes developing lncRNA-based treatments, administered via exosomes, which can be utilized to target specific tissues. If such pre-clinical investigations are successful, then ultimately, clinical trials will be required to evaluate the safety and effectiveness of using exo-lncRNAs in treating diabetic patients.

Further high throughput mechanistic studies are also warranted to elucidate the molecular pathways through which exo-lncRNAs are interrelated with diabetes and its consequences. This includes exploring interactions with key signaling pathways. Also, comparative analyses of the functions of particular exo-lncRNAs in T1DM and T2DM are essential for identifying distinct and common pathways involved in each type. Recognizing the differences may lead to tailored therapeutic strategies.

## Conclusion

Exo-lncRNAs play a significant role in the complex landscape of diabetes, providing key mechanistic insights. These molecules have been implicated in a variety of underlying mechanisms, ranging from the β-cell function and insulin resistance to their vascular complications. Exo-lncRNAs are special due to the remarkable stability of lncRNAs within exosomes, which allows them to facilitate cells’ crosstalk and influence critical metabolic and inflammatory pathways. Notably, exo-lncRNAs provide important clinical implications by dual promise. First, their presence in bodily fluids makes them attractive candidates for early, minimally invasive biomarkers of diabetes. Second, they are emerging as potential therapeutic targets, opening new avenues for the treatment of diabetes. Despite these advantages, significant limitations persist. For example, standardization of exo-lncRNAs isolation and analysis methods is still lacking, and sample variability continues to challenge reproducibility and clinical translation. Looking forward to future developments, such as high throughput single-cell exo-lncRNAs profiling and the creation of more reliable clinical assays, which are expected to accelerate their practical application. These innovations may lead to more personalized and proactive diabetes care. Therefore, ongoing studies are essential to fill the gap between these molecular discoveries and real-world clinical applications, ultimately aiming to improve outcomes for individuals affected by diabetes.

## Data Availability

No datasets were generated or analysed during the current study.

## Abbreviations

ABs: Apoptotic bodies
AD: Alzheimer’s disease
ADSC: Adipose-derived stem cell
ALK2: Activin receptor-like kinase 2
APCS: Adipocyte progenitor cells
BAT: Human brown adipose tissue
BBB: Blood–brain barrier
BDNF: Brain-derived neurotrophic factor
BMI: Body mass index
BMSCs: Bone marrow mesenchymal stem cells
CAD: Coronary artery disease
CDKN2A/B: Cyclin-dependent kinase inhibitor 2 A and 2B
CRC: Colorectal cancer
DFU: Diabetic foot ulcer
DMC: Diabetic microvascular complications
DN: Diabetic nephropathy
DR: Diabetic retinopathy
ECM: Extracellular matrix
ECs: Endothelial cells
EGFR: Epidermal Growth Factor Receptor
EMNVs: Extracellular vesicle-mimetic nanovesicles
EMT: Endothelial-mesenchymal transition
EPCS: Endothelial progenitor cells
ER: Endoplasmic reticulum
ERBB3: Erb-B2 Receptor Tyrosine Kinase 3
ESCRT: Endosomal sorting complex required for transport
ESKD: End-stage kidney disease
EVs: Extracellular vesicles
Exo-lncRNAs: Exosomal long noncoding RNAs
FOXA1: Forkhead Box A1
FOXO1: Forkhead box protein O1
GDF15: Growth Differentiation Factor 15
GDM: Gestational diabetes mellitus
GDM-M: GDM-related macrosomia
GSK: Glycogen Synthase Kinase
GSIS: Glucose-stimulated insulin secretion
HAMSCs: Human amniotic mesenchymal stem cells
HERC5: HECT and RLD Domain Containing E3 Ubiquitin Protein Ligase 5
HFMSCs: Hair follicle mesenchymal stem cells
HFD: High-fat diet
HG: High glucose
HG/PA: High glucose and palmitate
HMEC-1: Human dermal microvascular endothelial cells
Hoxa10: Homeobox A10
HRMECS: Human retinal microvascular endothelial cells
HRVEC: Human retinal vascular endothelial cells
HUVECS: Human umbilical vein endothelial cells
IDF: International Diabetes Federation
IFN-γ: Interferon-gamma
IGT: Impaired glucose tolerance
IL-10: Interleukin-10
IL-1β: Interleukin-1 beta
ILVs: Intraluminal vesicles
INS-1: Insulinoma-1 cells
INSIG1: Insulin-induced gene 1
Ins1: Insulin 1
Ins2: Insulin 2
Insr: Insulin receptor
IR: Insulin resistance
ISEV: International Society for Extracellular Vesicles
LC3B: Microtubule-Associated Protein 1 Light Chain 3 Beta
let-7: Lethal-7 miRNA
lincRNA: Intervening noncoding RNA
lncRNAs: Long noncoding RNAs
Lpl: Lipoprotein lipase
Mafa: Musculoaponeurotic Fibrosarcoma Oncogene Homolog A
MFGE8: Milk Fat Globule-EGF Factor 8
MIN6: Mouse Insulinoma 6 cells
miRNA: MicroRNA
mRNA: Messenger RNA
mTOR: Mechanistic Target of Rapamycin
MMP-X1: Matrix metalloproteinase-X1
MSCs: Mesenchymal stem cells
MVBs: Multivesicular bodies
MVs: Microvesicles
ncRNAs: Noncoding RNA
NET: Neutrophil extracellular trap
NF-κB: Nuclear Factor kappa-light-chain-enhancer of activated B cells
Nkx2.2: NK2 homeobox 2
Nkx6.1: NK6 Homeobox 1
NLRP3: NLR family pyrin domain containing 3
nt: Nucleotides
ox-LDL: Oxidized low-density lipoprotein
PAD: Peripheral arterial disease
PAQR3: Progestin and AdipoQ Receptor Family Member 3
PANDER: Pancreatic-derived factor
PBMC: Peripheral Blood Mononuclear Cell
PDR: Proliferative diabetic retinopathy
Pdx1: Pancreatic and Duodenal Homeobox 1
PI3K/AKT: Phosphoinositide 3-kinase/protein kinase B
PM: Plasma membrane
PTBP1: Polypyrimidine tract binding protein 1
PTEN: Phosphatase and Tensin Homolog
RBP4: Retinol-Binding Protein 4
RNases: Ribonucleases
ROS: Reactive oxygen species
SERPINB1: Serpin Family B Member 1
SIRT1: Sirtuin 1
SNAI1: Snail Family Transcriptional Repressor 1
SOX9: SRY-Box Transcription Factor 9
SREBP-1C: Sterol Regulatory Element-Binding Protein 1 C
STAT1: Signal Transducer and Activator of Transcription 1
STAT5: Signal Transducer and Activator of Transcription 5
STZ: Streptozotocin
T1DM: Type 1 diabetes mellitus
T2DM: Type 2 Diabetes Mellitus
TAC1: Tachykinin Precursor 1
TRADD: Tumor necrosis factor receptor type 1-associated death domain protein
TRAF3: TNF Receptor-Associated Factor 3
TRAF3IP2: TNF Receptor-Associated Factor 3 Interacting Protein 2
Twist1: Twist homolog 1
VEGF: Vascular Endothelial Growth Factor
VEGFA: Vascular endothelial growth factor A
VSMCs: Vascular smooth muscle cells
WHO: World Health Organization
Wnt: Wingless/Integration 1
XBP1: X-box Binding Protein 1

## Abbreviations of long noncoding RNAs

AC004862: Uncharacterized lncRNA transcript (Ensembl ID: AC004862)
AC006064.4: long noncoding RNA 006064.4
AC091729.2: Uncharacterized lncRNA transcript (Ensembl ID: AC091729.2)
AL133419.1: Uncharacterized lncRNA transcript (Ensembl ID: AL133419.1)
AL354872.2: Uncharacterized lncRNA transcript (Ensembl ID: AL354872.2)
ANRIL: Antisense non-coding RNA in the INK4 Locus
ANO3-AS1-201: ANO3 Antisense RNA 1 isoform 201
ATP8B3-3:1: Long non-coding RNA associated with ATPase phospholipid transporting 8B3 gene, transcript variant 3:1
Bhmt-AS: Betaine-homocysteine methyltransferase antisense RNA
Blinc1: Blimp1-associated long intergenic non-coding RNA 1
Blinc2: Blimp1-associated long intergenic non-coding RNA 2
Blinc3: β-cell long intergenic non-coding RNA 3
βFaar: β-cell function and apoptosis-associated RNA
C430049B03Rik: Clone 430049B03 RIKEN long non-coding RNA
CATG00000040069.1: Uncharacterized lncRNA transcript (Ensembl ID: CATG00000040069.1)
CCT5-212: Long non-coding RNA transcript associated with the CCT5 (Chaperonin Containing TCP1 Subunit 5) gene, transcript variant 212
COX17-2:3: Long non-coding RNA associated with Cytochrome c oxidase copper chaperone 17 gene, transcript variant 2:3
CTRB1-1:5: Long non-coding RNA associated with SCYL1 (SCY1 Like Pseudokinase 1)
DANCR: Differentiation antagonizes non-protein-coding RNA
DLX6-AS1: Distal-less homeobox 6 antisense RNA 1
EIF4ENIF1-1: Long non-coding RNA associated with Eukaryotic translation initiation factor 4E nuclear import factor 1 gene, transcript variant 1:1
ENSG00000269902: Ensembl transcript ID for [lncRNAs transcript)
ENSMUST00000181751.1: Mouse long non-coding RNA transcript (Ensembl transcript ID)
ENST0000046272: Ensembl transcript ID for lncRNAs transcript
ENST00000480633: Ensembl transcript ID for lncRNAs transcript
ENST00000588707.1: Uncharacterized lncRNA transcript (Ensembl ID: ENST00000588707.1)
ENST00000596839.1: Long non-coding RNA transcript ENST00000596839.1 (Ensembl transcript ID)
FAM190A-: Family with sequence similarity 190 member A3
FLJ16779: Long non-coding RNA FLJ16779
G006738: Uncharacterized lncRNA transcript (Ensembl ID: G006738)
G007039: Uncharacterized lncRNA transcript (Ensembl ID: G007039)
G042394: Uncharacterized lncRNA transcript (Ensembl ID: G042394)
GAS5: Growth arrest-specific 5
Gm10451: lncRNA gene model 10451
Gm16308 (Lnc03): Predicted gene 16308 , mouse long non-coding RNA 03
GSE61474_XLOC_014637: Uncharacterized lncRNA identified in the GEO dataset GSE61474 (XLOC_014637)
GTF3C5:1: Long non-coding RNA associated with GTF3C5 (General Transcription Factor IIIC Subunit 5)
H19: H19-Igf2 locus lncRNA
HI-LNC15: Human islet lncRNA 15
HOTAIR: HOX Transcript Antisense RNA
IK-208: Long non-coding RNA transcript associated with the IK (IKAROS family zinc finger 1) gene, transcript variant 208
KCNQ1OT1: KCNQ1 Overlapping Transcript 1
KLF3-AS1: Kruppel-Like Factor 3 Antisense RNA 1
LEGLTBC: Low expression in glucolipotoxicity-treated β-cells
LINC000958: Long intergenic non-protein coding RNA 958
LINC00473: Long intergenic non-protein coding RNA 473
LINC00960: Long Intergenic Non-Protein Coding RNA 960
LINC00968: Long intergenic non-protein coding RNA 968
LINC01018: Long Intergenic Non-Protein Coding RNA 1018
LINC02709-201: Long Intergenic Non-Protein Coding RNA 2709 isoform 201
linc00511: Long intergenic non-protein coding RNA 511
linc-p21: Long intergenic non-coding RNA-p21
Lnc13: Long non coding RNA 13
lnc17A: Long non-coding RNA 17A
lncAGER-1: Long noncoding RNA AGER-related transcript 1
lnc-HPS6-1:1: Long non-coding RNA associated with the HPS6 gene, transcript variant 1:1
lncRNA-1: Long non coding RNA 1
lncRNA-AGAP1-AS1: AGAP1 Antisense RNA 1
lncRNA-AFAP1-AS1: Actin Filament-Associated Protein 1 Antisense RNA 1
lncRNA BC200: Brain cytoplasmic RNA 200
lncRNA-p3134: a long non-coding RNA with transcript ID ENST00000545923
lncRNA PTGS2: lncRNA associated with the prostaglandin-endoperoxide synthase 2 gene
lnc-RXYLT1-3:2: Long non-coding RNA associated with Ribitol xylosyltransferase gene, transcript variant 3:2
lnc-TBC1D30-4:1: Long non-coding RNA associated with TBC1 domain family member 30 gene, transcript variant 4:1
lnc-ZFHX3-7:1: Long non-coding RNA ZFHX3 Antisense RNA 1, transcript variant 7:1
LOC100132249: Long non-coding RNA at chromosome 9q13
LY6E-DT-202: lncRNA LY6E divergent transcript 202
LYPLAL1-DT: LYPLAL1 divergent transcript
MALAT1: Metastasis-Associated Lung Adenocarcinoma Transcript 1
MEG3: lncRNA Maternally Expressed Gene 3
MEG3-230: Maternally Expressed Gene 3 isoform 230
MEG8: Maternally Expressed Gene 8
MIAT: Myocardial infarction-associated transcript
MSTRG.128697: Novel lncRNAs transcript ID from RNA-seq assembly
MSTRG.63013: Novel lncRNAs transcript ID from RNA-seq assembly
MSTRG.74858: Novel lncRNAs transcript ID from RNA-seq assembly
Mut-Reg1cp: Mutant form of the lncRNA Regenerating islet-derived 1 alpha pseudogene
MYL6-208: Long non-coding RNA transcript associated with the MYL6 (Myosin Light Chain 6) gene, transcript variant 208
NAMPT-AS: lncRNA NAMPT Antisense RNA 1
NDUFS7-204: Long non-coding RNA transcript associated with the NDUFS7 (NADH:Ubiquinone Oxidoreductase Subunit S7) gene, transcript variant 204
NONHSAG01135: Human long non-coding RNA transcript (NONCODE database identifier)
NR2F1-AS1: Nuclear Receptor Subfamily 2 Group F Member 1 Antisense RNA 1
NSA2-203: Long non-coding RNA transcript associated with the NSA2 (Ribosome Biogenesis Factor) gene, transcript variant 203
OIP5-AS1: Opa-interacting protein 5 antisense RNA 1
PANTR1: PAX8-associated non-coding transcript 1
PAX6-AS1: Paired Box Gene 6 antisense RNA 1
Pax6os1: Paired Box Gene 6 opposite strand 1
PLUTO: pdx1-associated lncRNA upstream transcript
POLG2-1:1: Long non-coding RNA associated with POLG2 (DNA Polymerase Gamma 2, Accessory Subunit)
PRINS: Psoriasis susceptibility-related RNA gene induced by stress
PVT1: Plasmacytoma variant translocation 1 long non-coding RNA
RPL13P5: lncRNA ribosomal protein L13 pseudogene 5
RPL35A-208: Long non-coding RNA transcript associated with the RPL35A (Ribosomal Protein L35a) gene, transcript variant 208
ROIT: RNA overlap interaction transcripts lncRNA
SCYL1-1:22: Long non-coding RNA associated with SCYL1 (SCY1 Like Pseudokinase 1)
SNHG16: Small nucleolar RNA host gene 16
SNHG5: Small nucleolar RNA host gene 5
SNHG7: Small Nucleolar RNA Host Gene 7 lncRNA
SNHG9: Small nucleolar RNA host gene 9
SRPK1-1:1: Long non-coding RNA associated with SRPK1 (SRSF Protein Kinase 1)
TCONS_00004187: Uncharacterized lncRNA transcript (RNA-seq assembled transcript ID: TCONS_00004187)
TFDP2-7:2: Long non-coding RNA associated with Transcription Factor Dp-2 gene, transcript variant 7:2
TUG1: Taurine Upregulated Gene 1
UCA1: Urothelial carcinoma-associated 1
XIST-224: X-inactive specific transcript isoform 224
XR_001778608.1: Predicted non-coding RNA transcript (RefSeq ID, NCBI)
XR_108954.2: Human long non-coding RNA transcript (RefSeq ID: XR_108954.2)
XR_880236.2: Predicted non-coding RNA transcript (RefSeq ID, NCBI)
ZBTB46-3:6: Long non-coding RNA associated with Zinc finger and BTB domain containing 46 gene, transcript variant 3:6
ZNF800-1:1: Long non-coding RNA associated with Zinc finger protein 800 gene, transcript variant 1:1
